# Prenatal valproic acid exposure in rodent models of autism: a systematic review of neurobehavioral physiological alterations

**DOI:** 10.3389/fphys.2026.1860596

**Published:** 2026-09-07

**Authors:** İbrahim Hakkı Çağıran, Dursun Alper Yilmaz

**Affiliations:** 1 Department of Nutrition and Dietetics, Muğla Sıtkı Koçman University, Mugla, Türkiye; 2 Department of Physiology-Medicine, Bursa Uludağ University, Institute of Health Sciences, Bursa, Türkiye; 3 Department of Nursing, Ağrı İbrahim Çeçen University, Faculty of Health Sciences, Agri, Türkiye

**Keywords:** autism spectrum disorder, microbiota–gut–brain axis, oxidative stress, prenatal exposure, rodent model, valproic acid

## Abstract

**Introduction:**

Rodent models based on prenatal valproic acid (VPA) exposure are widely used to investigate autism spectrum disorder (ASD)-related neurobehavioral and biological alterations. Over the past two years, the rapid expansion of VPA-based research incorporating advanced molecular, neuroimaging, electrophysiological, and microbiota analyses has generated a substantial but fragmented body of evidence.

**Methods:**

This systematic review aimed to synthesize findings from rodent studies published between January 2024 and November 2025 that employed prenatal VPA exposure, with particular emphasis on behavioral, neurobiological, inflammatory, microbiota–gut–brain axis, gene-expression, and histopathological outcomes. A structured search was conducted in PubMed, Scopus, Web of Science Core Collection, Embase, and PsycINFO, supplemented by manual screening in Google Scholar. Controlled vocabulary and free-text terms related to ASD, VPA, and rodent models were combined. Original *in vivo* studies administering VPA during gestation and reporting behavioral and/or biological outcomes were included. Because of substantial heterogeneity in VPA dose, administration route, gestational timing, species, strain, sex, and outcome domains, findings were synthesized narratively. Risk of bias was assessed using the SYRCLE risk-of-bias tool, and reporting completeness was evaluated using the ARRIVE 2.0 guidelines.

**Results:**

Sixty-six eligible prenatal VPA studies were included. Most administered a single intraperitoneal dose of 500 or 600 mg/kg around embryonic day 12–12.5. Across studies, prenatal VPA exposure was consistently associated with ASD-like phenotypes, including reduced sociability, impaired social novelty preference, repetitive behaviors, anxiety-like traits, and cognitive deficits. Frequently reported biological alterations included oxidative stress, neuroinflammation involving NF-κB and NLRP3 signaling, neurotransmitter dysregulation, impaired synaptic plasticity, microbiota-related changes, and region-specific brain abnormalities.

**Discussion:**

Recent prenatal VPA studies therefore extend beyond classical behavioral characterization by integrating neuroimmune, oxidative, microbiota-related, transcriptomic, electrophysiological, and histopathological findings. However, substantial methodological heterogeneity and incomplete reporting of randomization, blinding, animal-flow, and litter-level procedures limit cross-study comparability, reproducibility, and translational interpretation.

## Graphical Abstract

**Figure:**
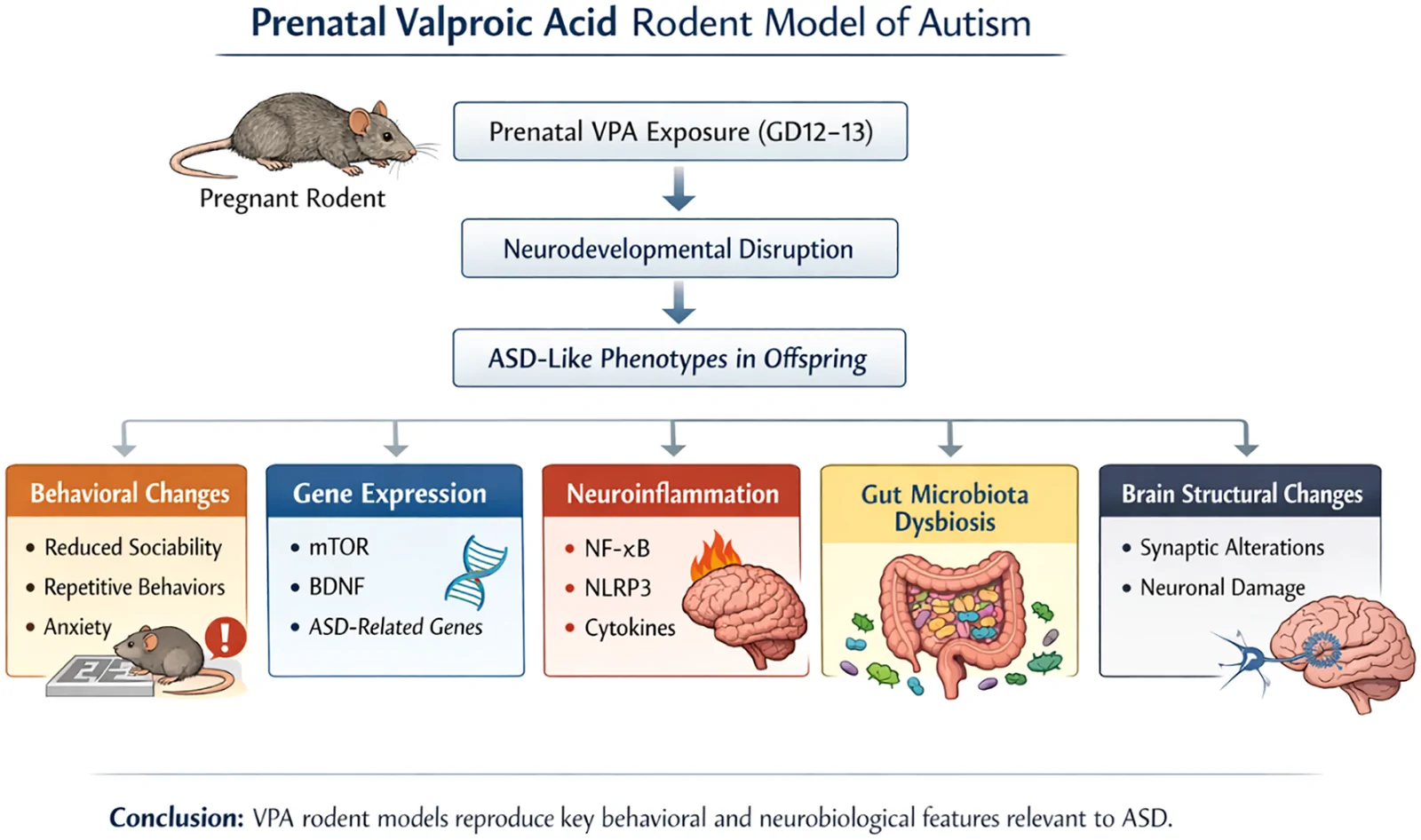
Prenatal valproic acid exposure in rodents consistently induces autism-like behavioral and neurobiological alterations. Recent studies highlight inflammation, microbiota disruption, and gene-expression changes.

## Introduction

Autism Spectrum Disorder (ASD) is a heterogeneous neurodevelopmental condition characterized by persistent impairments in social communication and interaction, together with restricted and repetitive behaviors (American Psychiatric Association, 2013; Volkmar et al., 2014; Hodges et al., 2020). Clinical manifestations, including delayed language, motor and cognitive development, altered responsiveness, hyperactivity, restricted interests, and stereotypic or self-injurious behaviors, reflect the substantial phenotypic variability observed among individuals with ASD (Elder et al., 2008; American Psychiatric Association, 2013; Volkmar et al., 2014; Mehra et al., 2022). Although advances in diagnostic approaches have improved recognition of ASD, its etiology remains complex and incompletely understood. Current evidence indicates that ASD arises from the interplay of genetic susceptibility, epigenetic regulation, and environmental influences that affect early neurodevelopmental processes (Chaste and Leboyer, 2012; Fett-Conte et al., 2015; Eshraghi et al., 2018). Genetic mutations, chromosomal abnormalities, and copy-number variations, together with prenatal environmental exposures such as chemical agents and maternal stressors, may disrupt fetal brain development (Fett-Conte et al., 2015). Furthermore, epigenetic mechanisms, including DNA methylation and histone modifications, contribute to alterations in gene regulation and neurodevelopmental trajectories associated with ASD vulnerability (Eshraghi et al., 2018).

Despite substantial progress in understanding ASD mechanisms, available interventions remain primarily focused on symptom management rather than modifying core disease mechanisms. Therefore, the development and refinement of valid preclinical models remain essential for investigating ASD-related biological pathways, identifying potential therapeutic targets, and understanding the complex interactions between molecular, cellular, and behavioral alterations. Rodent models have become particularly valuable in ASD research because they allow controlled investigation of developmental processes and experimental manipulation of genetic and environmental risk factors.

Several rodent models have been developed to investigate ASD pathophysiology and are broadly categorized into genetic and environmental models. Genetic models commonly target ASD-associated genes, including Shank3, Cntnap2, Fmr1, Mecp2, and Nlgn3, enabling investigation of specific molecular mechanisms involved in synaptic development and neuronal signaling. Environmental models include maternal immune activation (MIA), prenatal exposure to valproic acid (VPA), propionic acid administration, and maternal stress paradigms, which represent non-genetic developmental risk factors associated with ASD. Each model captures distinct aspects of ASD-related phenotypes and presents specific advantages and limitations regarding construct, face, and predictive validity. Consequently, no single animal model fully reproduces the clinical heterogeneity and biological complexity of ASD, and model selection should be guided by the specific research question.

Among environmental ASD models, prenatal exposure to VPA, a short-chain fatty acid also known as 2-propylpentanoic acid, represents one of the most extensively characterized and widely used experimental approaches (Hrabovska and Salyha, 2016; El-Ansary et al., 2018; Nicolini and Fahnestock, 2018; Baker et al., 2020; Zohny et al., 2023; Zarate-Lopez et al., 2024). VPA is clinically used as an antiepileptic and mood-stabilizing agent; however, prenatal exposure has been associated with congenital anomalies, cognitive impairments, and increased risk of ASD-related features in offspring (Zohny et al., 2023; Zarate-Lopez et al., 2024). Mechanistically, VPA induces broad epigenetic modifications through histone deacetylase (HDAC) inhibition, influencing neurodevelopmental processes and gene regulation (Nicolini and Fahnestock, 2018). Consistent with clinical observations, rodents prenatally exposed to VPA exhibit several ASD-like phenotypes, including reduced sociability, impaired communication, anxiety-like behaviors, repetitive behaviors, and cognitive alterations, supporting the usefulness of this model for investigating selected ASD-related features while highlighting its partial translational relevance (Hrabovska and Salyha, 2016; Nicolini and Fahnestock, 2018; Zohny et al., 2023; Zarate-Lopez et al., 2024).

The biological effects of prenatal VPA exposure involve multiple interconnected neurodevelopmental pathways. Through indirect inhibition of GSK-3β, VPA activates the Wnt/β-catenin pathway, which plays an important role in cortical patterning and neural progenitor differentiation (Hall et al., 2002; Chenn, 2008; Jung et al., 2008). Enhanced β-catenin signaling influences Ras–ERK–p21-mediated maturation processes (Jung et al., 2008), while increased GABAergic activity resulting from inhibition of GABA transaminase and activation of glutamic acid decarboxylase may disrupt excitatory–inhibitory balance during critical developmental periods (Sernagor, 2010; Nicolini and Fahnestock, 2018). These molecular alterations provide a mechanistic basis for the behavioral abnormalities, synaptic dysfunction, and neuropathological changes observed in VPA-exposed rodents.

In addition to neurodevelopmental and molecular alterations, emerging evidence suggests that prenatal VPA exposure may influence the microbiota–gut–brain axis, which has increasingly been implicated in ASD pathophysiology. Recent studies in VPA-induced rodent models have reported alterations in gut microbial composition, reduced microbial diversity, intestinal barrier dysfunction, changes in microbial-derived metabolites such as short-chain fatty acids, and dysregulation of inflammatory signaling pathways (Liu et al., 2024; Prince et al., 2024; Yang et al., 2024; Ay et al., 2025; Fang et al., 2025; Wu et al., 2025). These findings suggest that microbiota-related mechanisms may interact with neuroimmune and neurodevelopmental processes through bidirectional communication between the gastrointestinal system and the central nervous system, potentially contributing to ASD-like behavioral phenotypes observed following prenatal VPA exposure (Liu et al., 2024; Prince et al., 2024; Ay et al., 2025; Fang et al., 2025). Therefore, integrating microbiota-related alterations with molecular and behavioral findings may provide a broader understanding of the biological mechanisms underlying the prenatal VPA model.

Although previous reviews have provided important perspectives on prenatal VPA exposure, these studies have primarily focused on model validity, classical behavioral phenotyping, neurobiological mechanisms, and general characterization of VPA-induced ASD features (Nicolini and Fahnestock, 2018; Mehra et al., 2022; Zarate-Lopez et al., 2024). Zarate-Lopez et al. (2024) provided a comprehensive overview of the historical development, behavioral characteristics, and general validity of the prenatal VPA model. However, recent experimental studies have increasingly incorporated advanced approaches, including transcriptomics, multi-omics analyses, neuroimmune profiling, microbiota investigations, and circuit-level assessments, which have substantially expanded the mechanistic understanding of this model. Therefore, an updated synthesis integrating these emerging biological domains is warranted.

Accordingly, this systematic review aimed to critically synthesize recent rodent studies published between 2024 and 2025 investigating prenatal VPA exposure and ASD-related outcomes. Specifically, this review integrates behavioral, inflammatory, oxidative stress-related, microbiota-related, gene-expression, molecular, and neuroanatomical findings to provide an updated perspective on the translational relevance, methodological limitations, and emerging mechanistic insights of the prenatal VPA model.

## Materials and methods

### Search strategy

A comprehensive search was conducted in PubMed (MEDLINE), Scopus, Web of Science Core Collection, Embase, and PsycINFO to identify experimental rodent studies investigating VPA-induced autism models. Google Scholar was additionally screened to identify potentially relevant studies not indexed in the primary databases and to capture grey literature.

The search strategy included a combination of controlled vocabulary terms and free-text keywords related to autism spectrum disorder (ASD), valproic acid, and rodent models. The main search terms included “autism,” “autism spectrum disorder,” “valproic acid,” “VPA,” “rat model,” “mouse model,” and “rodent model,” combined using Boolean operators. All databases were searched for studies published between January 2024 and November 2025 to capture recent evidence.

### Eligibility criteria

Eligibility criteria were defined according to the PICOS framework. The population included rodent models (rats and mice) used in experimental studies of ASD-related phenotypes. The intervention was prenatal exposure to VPA, regardless of dose, administration route, or gestational timing. The comparator consisted of control groups without prenatal VPA exposure. Eligible outcomes included behavioral, molecular, inflammatory, microbiota-related, gene-expression, and histopathological alterations associated with prenatal VPA exposure. Only original *in vivo* experimental animal studies were included.

Studies were excluded if they were descriptive studies, letters, book chapters, conference proceedings, narrative reviews, systematic reviews, or studies focusing solely on nutritional or adjunctive interventions without the use of VPA-induced ASD modeling.

### Study selection process

Study selection was performed according to the Preferred Reporting Items for Systematic Reviews and Meta-Analyses (PRISMA 2020) guidelines. Two reviewers independently screened titles and abstracts, followed by full-text assessment according to predefined eligibility criteria.

**Figure 1:**
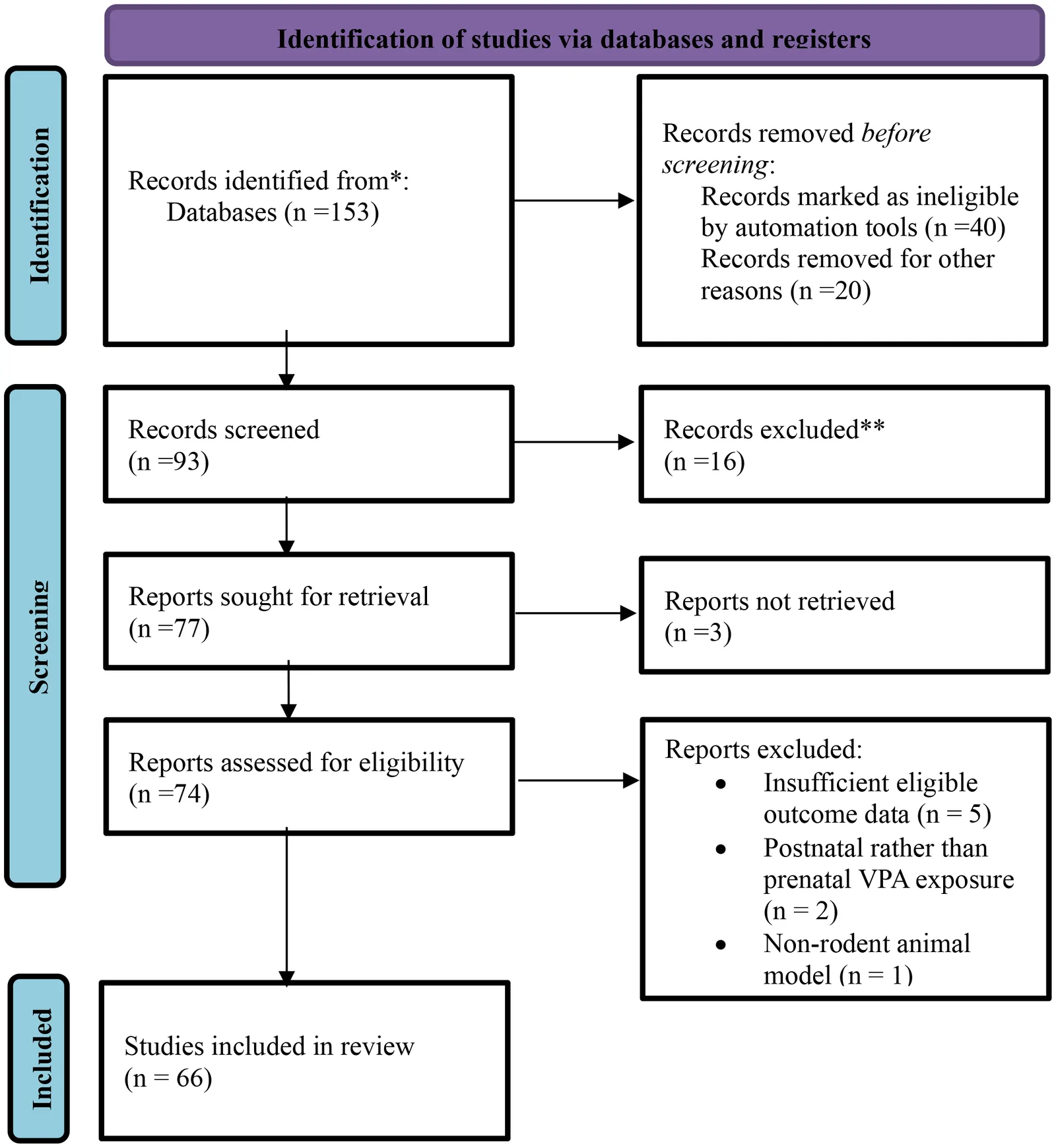
PRISMA flow diagram of article selection.

Disagreements between reviewers were resolved through discussion and consensus. The complete selection process is illustrated in the PRISMA flow diagram (**Figure 1**).

Because several research groups published multiple articles derived from the same experimental cohort, PRISMA 2020 terminology distinguishing “studies” (unique experimental cohorts) and “reports” (individual publications derived from those cohorts) was applied. Following duplicate removal and full-text eligibility assessment, 66 eligible prenatal VPA reports were included in the systematic review.

### Data extraction

Data extraction was performed independently by two reviewers using a predefined data extraction form. The extracted information included the first author and publication year, animal species and strain, sex, sample size, VPA dose, administration route, and gestational timing of exposure. In addition, data related to behavioral outcomes, molecular and inflammatory findings, microbiota-related alterations, gene-expression changes, and histopathological findings were extracted and systematically categorized for synthesis.

### Risk of bias assessment

The methodological quality and risk of bias of included studies were assessed using the SYRCLE risk-of-bias tool for animal intervention studies.

The assessment included domains related to selection bias, performance bias, detection bias, attrition bias, and reporting bias. Detailed results of the SYRCLE assessment are presented in **Supplementary Table 1**. In addition to the SYRCLE risk-of-bias assessment, the methodological reporting quality of the included animal studies was evaluated in accordance with key recommendations from the ARRIVE guidelines. Reporting domains assessed included experimental design, animal characteristics, intervention details, and outcome reporting, where applicable.

### Data synthesis

Due to substantial heterogeneity among included studies regarding VPA dose, administration route, gestational timing, animal strain, sex, and evaluated outcomes, quantitative meta-analysis was not performed.

Instead, findings were synthesized narratively and grouped according to major outcome domains, including behavioral alterations, inflammatory responses, microbiota-related changes, gene-expression alterations, molecular mechanisms, and histopathological findings.

## Results

A total of 66 studies met the inclusion criteria and demonstrated diverse behavioral, molecular, inflammatory, microbiota-related, and histopathological effects following prenatal VPA exposure (**Tables 1**, **2**).

**Table 1:** General characteristics of included studies.

First author, year [ref]	Species/strain	Sex	n	Dose (mg/kg)	Route	GD	Injections	Main outcome domains
Zhu et al. (2025)	Sprague–Dawley rats	M/F	27	500	i.p.	E12.5	Single	Behavior, growth, gene expression
Campos et al. (2025)	Wistar rats	M/F	18	600	i.p.	E12	Single	Social behavior, oxidative & inflammatory markers
Tarahomi et al. (2025)	Rats (N.S.)	M	14	500	i.p.	E12.5	N.S.	Nociception, anxiety, oxidative stress, cognition
Benli et al. (2025)	Wistar rats	M	40	600	i.p.	E12.5	Single	Sensorimotor, social behavior, synaptic function
Fang et al. (2025)	C57BL/6J mice	M/F	45	600	i.p.	E12.5	N.S.	Microbiota, metabolomics, social deficits
Roh et al. (2024)	Sprague–Dawley dams	M/F	N.S.	500	i.p.	E12.5	Single	Neuronal structure/function, firing properties
Mohamed et al. (2025)	Wistar rats	M	N.S.	600	i.p.	E12.5	Single	ER stress, autophagy, microglia, behavior
Chu et al. (2025)	C57BL/6J mice	M	20	500	i.p.	E12.5	Single	Fear conditioning, social behavior, synaptic function
Hosseini et al. (2025)	Wistar rats	M	56	600	i.p.	E12.5	Single	Anxiety, repetitive behavior, GSK-3β, BDNF
Elsherif et al. (2025)	Albino Wistar pups	M	54	600	s.c.	E13	Single	Social behavior, oxidative stress, PI3K-mTOR
Mansour and Kulesza, 2024)	Albino rats	M	20	600	i.p.	E12	N.S.	Cerebellar histology, apoptosis, neuronal degeneration
Cezar et al. (2025)	Wistar rats	M	50	400	i.p.	E12.5	N.S.	Dopamine pathways, stereotypy, hyperactivity
Lian et al. (2025)	Sprague–Dawley rats	M/F	107	300–800	N.S.	E12.5	N.S.	Developmental trajectory, sex differences
Jiang et al. (2025)	SD rats	M	30	500	i.p.	E12.5	Single	Neuroinflammation, behavior, microglia
Farbin et al. (2024)	SD rats	M/F	40	600	i.p.	E12.5	Single	Oxidative stress, TNF-α, IL-1β, IL-6, cognition
Ibáñez-Sandoval et al. (2024)	Long-Evans rats	M/F	40	600	i.p.	E12	Single	Brain volume, ACC, hippocampus development
Sun et al. (2024)	C57BL/6J mice	M/F	56	150	i.p.	E13.5	Single	Hippocampal neurogenesis
Zahedi et al. (2024)	SD rats	M	N.S.	500	i.p.	E12.5	Single	Sensory processing, interneurons
Ohnami et al. (2024)	Fmr1-KO mice	M	N.S.	500	i.p.	E12.5	N.S.	Prosocial brain activation, repetitive behaviors
He et al. (2025)	Wistar rats	M/F	20	600	i.p.	E12.5	Single	Serotonin, neurotransmitters, thalamus morphology
Ay et al. (2025)	SD rats	M/F	30	500	i.p.	E12–13	Single	Microbiota, SCFA metabolism, inflammation
Alnakhli et al. (2024)	Wistar rats	M	10	600	i.p.	E12.5	Single	Oxidative stress, inflammation, PI3K/Akt/GSK3β
Tamaoki et al. (2024)	SD rats	M/F	15	600	i.p.	E12.5	N.S.	Auditory processing, speech discrimination
Liu et al. (2024)	C57BL/6J mice	N.S.	16	500	i.p.	E12.5	Single	Microglia–PNN balance
Mohammadkhani et al. (2024)	Wistar rats	M/F	48–64	500	i.p.	E12.5	Single	Exercise response, LTP
Palanivelu et al. (2024)	Mice	M	N.S.	500	i.p.	E12.5	Single	GABAA receptor alterations
Lu et al. (2024)	SD rats	M	48	500	i.p.	E12.5	Single	Oxidative stress, mitochondrial dysfunction
Prince et al. (2024)	C57BL/6J mice	M	24–39	600	i.p.	E12.5	N.S.	Microglial morphology, anxiety, social behavior
Saavedra-Bonilla et al. (2024)	Wistar rats	M	N.S.	600	i.p.	E12.5	Single	ROS, oxidative stress, interneuron loss
King et al. (2024a)	SD rats	M	15	800	Oral	E10 & E12	Two doses	Auditory nuclei, brainstem connectivity
Yang et al. (2025)	C57BL/6J mice	M/F	20	N.S.	i.p.	E12.5	Single	Housing-dependent social behavior changes
King et al. (2024b)	Long-Evans rats	M/F	132	600	i.p.	E12	Single	PV+ neuron changes, cognitive function
King et al. (2024c)	Long-Evans rats	M/F	49	600	i.p.	E12	Single	BDNF, cognition, antioxidant enzymes
Rahdar et al. (2024)	Wistar rats	M	65	500	i.p.	E12.5	Single	Serotonin 5-HT7 modulation, electrophysiology
Yang et al. (2024)	Wistar rats	M	60	600	i.p.	E12.5	Single	Gut structure, gut neurotransmitters, inflammation
Horata et al. (2024)	Wistar rats	M	30	600	i.p.	E12.5	Single	Thalamic neurons, hemispheric volume
Farrag et al. (2024)	SD rats	M	24	500	i.p.	E12.5	Single	TLR4/NF-κB/NOX-2 signaling, inflammation
Mokhtar Tawfeek et al. (2025)	Swiss albino mice	M/F	15	600	i.p.	E12.5	N.S.	Behavior, cognition, brain histopathology, neurodegeneration
Chen S et al. (2024)	Wistar rats	M	8	600	i.p.	E12.5	N.S.	Anxiety, repetitive behavior, social behavior, synaptic & mitochondrial ultrastructure
Liao et al. (2024)	Sprague–Dawley rats	M	30	600	i.p.	E12.5	N.S.	Sortilin/PGRN signaling, inflammation, dendritic spines, behavior
Reza naghdi et al. (2024)	Wistar rats	M	8	600	i.p.	E12.5	Single	Anxiety, repetitive behavior, oxidative stress, ERK1/2 signaling
Wang et al. (2024b)	C57BL/6J & nNOS-GFP mice	N.S.	30	600	i.p.	E12.5	N.S.	Social behavior, communication, stereotypy, AMPA/nNOS signaling
Godavarthi et al. (2024)	C57BL/6J, PV-Cre, CCK-IRES-Cre, VGAT-Cre mice	M/F	9	300	i.p.	E12.5	Single	Neurotransmitter switching (GABA→Glu), social & repetitive behavior
Wang et al. (2024a)	B6 mice	M	N.S.	400	s.c.	E14	N.S.	Social behavior, anxiety, depression, synaptic function
Sun et al. (2024)	Sprague Dawley rats	M	50	400	i.p.	E13.5	Single	Microglial activation, sociability, exploration
Afshari et al. (2024)	Wistar rats	N.S.	19	600	i.p.	E12.5	Single	Social vs. non-social preference, anxiety
Sivayokan et al. (2024)	Long-Evans rats (dams)	M/F	93	600	i.p.	E12	Single	mPFC/ACC/hippocampal volumes, sex-specific changes
Souza et al. (2024)	Sprague–Dawley rats	M/F	20–40	600	i.p.	E12	N.S.	Social behavior, grooming, communication, DA neuron activity
Chen B et al. (2024)	Sprague–Dawley rats	M	14	500	i.p.	E12.5	Single	Social orientation, memory, hippocampal oscillations
Neelotpol et al. (2024)	CD-1 mice	M/F	52	250	i.p.	E12.5	Single	δ-catenin, dendritic branching, synaptic AMPA, USV
Yu et al. (2024)	Sprague–Dawley rats	M	15	200/300/500	i.p.	E12.5	N.S.	Kv10.2, Akt/mTOR signaling
Liu et al. (2024)	C57BL/6J (B6J) & BTBR mice	M	30	600	i.p.	E12.5	N.S.	Gut barrier, cytokines, HDAC1, behavior
Wu et al. (2025)	Wistar rats	M	12	450	i.p.	E12.5	N.S.	Maternal intestinal health, offspring gut function
Miao et al. (2024)	Sprague–Dawley rats	M	15	500	i.p.	E12.5	Single	ROS, mitochondrial function, social & cognitive behavior
Lu et al. (2024)	Sprague Dawley rats	F	100	500	i.p.	E12.5	Single	Oxytocin/OXTR in hippocampus & PFC, social & anxiety behavior
Finszter et al. (2024)	C57BL mice	M/F	20	500	i.p.	E13.5	N.S.	Dopaminergic projections, NAc/CPu TH changes, reward circuitry

N.S., not specified; s.c., subcutaneous; i.p., intraperitoneal.

**Table 2:** Behavioral outcomes summary .

First author, year [ref]	Social behavior	Anxiety-like	Repetitive behavior	Cognition/memory	Locomotor	Notes
Zhu et al. (2025)	↓ social interaction	↓ center time	N.S.	↓ exploration	N.S.	Extensive gene expression changes
Campos et al. (2025)	↓ social approach	N.S.	N.S.	N.S.	N.S.	Sex-specific oxidative changes
Tarahomi et al. (2025)	N.S.	↑ anxiety	N.S.	↓ learning	N.S.	↑ nociceptive threshold
Benli et al. (2025)	↓ sociability	N.S.	N.S.	↓ recognition	N.S.	↓ fEPSP slope
Fang et al. (2025)	↓ sociality	↑ anxiety	↑ stereotypy	N.S.	↓ movement	Microbiota shifts
Roh et al. (2024)	↓ social interaction	N.S.	N.S.	N.S.	N.S.	Sex-specific neuronal morphology
Mohamed et al. (2025)	Impaired social	↑ anxiety	↑ self-grooming	N.S.	N.S.	ER stress & autophagy
Hosseini et al. (2025)	↓ sociability	↑ anxiety	↑ stereotypy	N.S.	↑ sniffing	↑ GSK-3β, ↓ BDNF
Elsherif et al. (2025)	↓ social	↑ anxiety	N.S.	N.S.	↑ locomotion	PI3K-mTOR dysregulation
Mansour and Kulesza, (2024)	Impaired social	↑ anxiety	N.S.	N.S.	N.S.	Cerebellar degeneration
Cezar et al. (2025)	↓ social	↑ anxiety	↑ stereotypy	↓ memory	↑ locomotion	↓ dopamine
Lian et al. (2025)	↓ social	N.S.	↑ fixation	N.S.	N.S.	Females > social than males
Jiang et al. (2025)	↓ social	N.S.	↑ stereotypy	Impaired	N.S.	NF-κB activation
Farbin et al. (2024)	↓ social	↑ anxiety	↑ grooming	↓ learning	N.S.	Oxidative + inflammatory markers
Ibáñez-Sandoval et al. (2024)	Social deficits	Anxiety-like	N.S.	N.S.	N.S.	Brain structural changes
Sun et al. (2024)	↓ social	N.S.	N.S.	N.S.	N.S.	↓ hippocampal neurogenesis
Zahedi et al. (2024)	↓ social	↑ anxiety	↑ stereotypy	N.S.	↓ exploration	Atypical somatosensory processing
Ohnami et al. (2024)	↓ social	N.S.	↑ repetitive	N.S.	N.S.	Prosocial neuron activation
He et al. (2025)	↓ interaction	N.S.	N.S.	N.S.	N.S.	↑ serotonin, unchanged GABA/GLU
Ay et al. (2025)	↓ social	↑ anxiety	↑ stereotypy	N.S.	↑ activity	Microbiota & SCFA changes
Alnakhli et al. (2024)	↓ social	↑ anxiety	↑ repetitive	N.S.	N.S.	PI3K/Akt/GSK3β + JAK/STAT
Tamaoki et al. (2024)	Impaired	N.S.	N.S.	Impaired auditory learning	↑ reflex freezing	Auditory brainstem deficits
Liu et al. (2024)	↓ social	N.S.	N.S.	N.S.	N.S.	↑ PNNs in hippocampus/visual cortex
Mohammadkhani et al. (2024)	Sex-differences	N.S.	N.S.	↑ LTP (male with exercise)	N.S.	Exercise-modulated plasticity
Palanivelu et al. (2024)	↓ social	↑ anxiety	↓ stereotypy	Impaired	N.S.	GABAA-ρ3 reduction
Lu et al. (2024)	↓ social	↑ anxiety	↑ grooming	↓ recognition	N.S.	Oxidative + mitochondrial stress
Saavedra-Bonilla et al. (2024)	↓ social	↑ anxiety	↑ stereotypy	N.S.	N.S.	PV interneuron loss
King et al. (2024a)	↓ social	N.S.	N.S.	↓ auditory discrimination	N.S.	Brainstem projection loss
Yang et al. (2025)	Housing-dependent ↓ social	N.S.	↑ stereotypy	N.S.	N.S.	Critical period sensitivity
Chu et al. (2025)	↓ social	↑ anxiety	↑ grooming	↓ learning	N.S.	AMPK–mTOR pathway
King et al. (2024b)	↓ social	N.S.	N.S.	Impaired	N.S.	PV+ neuron alterations
King et al. (2024c)	↓ social	N.S.	N.S.	↓ cognition	↑ activity	↑ BDNF with exercise
Rahdar et al. (2024)	↑ social after LP-211	↓ anxiety	↓ stereotypy	↑ learning	N.S.	5-HT7R-mediated rescue
Yang et al. (2024)	↓ social	↑ anxiety	↑ repetitive	↓ learning	↓ activity	Gut neurotransmitter imbalance
Horata et al. (2024)	↓ social	N.S.	N.S.	↓ memory	N.S.	↓ thalamic neurons
Farrag et al. (2024)	↓ social	↑ anxiety	↑ stereotypy	N.S.	N.S.	TLR4–NF-κB activation
Mokhtar Tawfeek et al. (2025)	↓ social	↑ anxiety	↑ stereotypy	↓ memory	↑ locomotion	Neuronal degeneration
Chen et al. (2024)	↓ social interaction & novelty preference	↑ anxiety	↑ stereotyped/repetitive	↓ spatial exploration	↓ activity	Synaptic, mitochondrial, lysosomal abnormalities
Liao et al. (2024)	Impaired social behavior	↑ anxiety	↑ repetitive behavior	N.S.	N.S.	↑ sortilin, ↓ PGRN, ↑ microglia
Reza naghdi et al. (2024)	↓ social interaction	↑ anxiety	↑ grooming/repetitive	↓ novel object detection	N.S.	↑ MDA, ↓ GPX, ↓ SOD, ↓ TAC, ERK1/2 disruption
Wang et al. (2024b)	Abnormal social interaction	Anxiety-like traits	Stereotypical/repetitive	N.S.	N.S.	↓ nNOS, altered AMPA-mediated transmission
Godavarthi et al. (2024)	↓ social (males)	N.S.	↑ stereotypy (both sexes)	N.S.	N.S.	GABA→Glu shift in PV/CCK interneurons
Wang et al. (2024a)	↓ social activity	↑ anxiety	↑ repetitive behavior	N.S.	N.S.	Depression-like traits, synaptic dysfunction
Sun et al. (2024)	↓ sociability & interaction	N.S.	N.S.	N.S.	↓ center time, ↓ exploration	Microglial activation, tail deformity
Afshari et al. (2024)	↓ social interaction time	↑ anxiety	N.S.	N.S.	N.S.	Prefers non-social stimuli
Sivayokan et al. (2024)	Social deficits	N.S.	N.S.	↓ cognitive flexibility	N.S.	Regional volume irregularities (mPFC, ACC, hippocampus)
Souza et al. (2024)	↓ social interaction (rescued by modulator)	N.S.	↑ stereotypy (reduced by drug)	↓ cognition (improved by drug)	Hyperactivity	GABAA α5 modulation, DA hyperactivity unchanged
Chen et al. (2024)	↓ social orientation	N.S.	N.S.	↓ memory	N.S.	Disrupted theta–gamma coupling, oscillatory patterns
Neelotpol et al. (2024)	N.S.	N.S.	Altered USV patterns	N.S.	N.S.	↓ δ-catenin, ↓ AMPA, dendritic changes
Prince et al. (2024)	↓ social & cognition	↑ anxiety	↑ stereotypy	↓ cognitive performance	N.S.	Microbiota & gut permeability changes
Yu et al. (2024)	N.S.	N.S.	N.S.	N.S.	N.S.	Primarily molecular (Kv10.2, Akt/mTOR), no detailed behavior reported
Liu et al. (2024)	Behavioral dysfunction (autistic-like)	N.S.	↑ repetitive traits	N.S.	N.S.	Gut barrier disruption, cytokine elevation
Wu et al. (2025)	N.S.	N.S.	N.S.	N.S.	N.S.	Focus on GI function-no detailed behavioral testing
Miao et al. (2024)	↓ social interaction	N.S.	N.S.	↓ cognitive performance	N.S.	Nanoparticle-rapamycin rescue, mitochondrial/ROS modulation
Liu et al. (2024)	Social interaction deficits	↑ anxiety	↑ stereotypic sexual behavior	N.S.	N.S.	↑ oxytocin/OXTR, impaired synaptic plasticity
Finszter et al. (2024)	N.S.	N.S.	N.S.	N.S.	N.S.	Main focus on DA pathway development & forebrain circuit differentiation

N.S., not specified. ↑ indicates an increase, and ↓ indicates a decrease.

### Animal species and strains

Among the 66 eligible prenatal VPA studies, rats were used in 47 studies (71.2%), whereas mice were used in 19 studies (28.8%). Wistar rats were the most frequently used strain (n = 23), followed by Sprague–Dawley rats (n = 19) and Long–Evans rats (n = 4); the rat strain was not specified in one study. Among mouse models, C57BL/6-lineage mice were the most commonly used (n = 12), followed by Swiss mice (n = 2) and CD-1 mice (n = 2). BALB/c mice and ICR mice were each used in one study, while the mouse strain was not specified in one study. Overall, the predominance of Wistar and Sprague–Dawley rats highlights the continued reliance on rat models for investigating the neurodevelopmental and behavioral consequences of prenatal VPA exposure.

### VPA dosage

Across the 66 eligible prenatal VPA studies, eight different VPA dose levels were identified, ranging from 150 to 800 mg/kg. The most frequently administered dose was 600 mg/kg, reported in 34 studies (51.5%), followed by 500 mg/kg in 24 studies (36.4%). A dose of 400 mg/kg was used in three studies (4.5%), whereas doses of 150, 250, 300, 450, and 800 mg/kg were each reported in one study (1.5%). Overall, 58 of the 66 studies (87.9%) administered VPA at either 500 or 600 mg/kg, indicating that these doses represent the current standard experimental paradigm for prenatal VPA-induced ASD models. Nevertheless, variation in VPA dosage may influence the severity of neurodevelopmental alterations and contribute to differences in behavioral, molecular, and neurobiological outcomes across studies.

### Route of administration

Three routes of prenatal VPA administration were identified: intraperitoneal, subcutaneous, and oral administration. Intraperitoneal injection was overwhelmingly the predominant method, whereas subcutaneous and oral administration were used only occasionally. Differences in administration route may affect systemic bioavailability, peak plasma concentrations, placental transfer, and fetal VPA exposure, thereby representing an additional source of variability among experimental protocols.

### Timing of prenatal VPA exposure

Prenatal VPA administration was performed between embryonic day (E) 10 and E13.5, with E12.5 being the most frequently selected time point. Fewer studies administered VPA on E11, E12, E13, or E13.5, whereas one study administered VPA on both E10 and E12, and another reported administration within the E12–E13 interval. The predominance of E12.5 reflects its widespread use as a critical developmental window during which prenatal VPA exposure disrupts neurogenesis, neuronal migration, cortical organization, and subsequent neural circuit maturation, thereby producing ASD-like phenotypes in rodent offspring (Tartaglione et al., 2019; Dorsey et al., 2024; Zarate-Lopez et al., 2024). Differences in the timing of prenatal exposure may also contribute to variability in the behavioral, neuropathological, and molecular characteristics reported across studies.

## Effects of valproic acid on behavior

Despite marked variation in dose, strain, sex, and gestational timing, ASD-relevant behavioral abnormalities (particularly reduced sociability and increased repetitive behaviors) were commonly reported across studies. Zhu et al. reported impaired exploratory activity and disrupted social interactions based on OFT and TCST scores in 27 rats (Zhu et al., 2025). Another study found increased time spent in dark areas, elevated self-motor activity, and hyperactivity (Godavarthi et al., 2024). Numerous studies demonstrated reduced social approach in both rats and mice (Afshari et al., 2024; Alnakhli et al., 2024; Chen et al., 2024; Chen et al., 2024; Farbin et al., 2024; Farrag et al., 2024; Godavarthi et al., 2024; Horata et al., 2024; Kurahashi et al., 2024; Liao et al., 2024; Liu et al., 2024; Liu et al., 2024; Lu et al., 2024; Meng et al., 2024; Miao et al., 2024; Noshadian et al., 2024; Ohnami et al., 2024; Prince et al., 2024; Qiu et al., 2024; Reza naghdi et al., 2024; Souza et al., 2024; Sun et al., 2024; Sun et al., 2024; Wang et al., 2024b; Wang et al., 2024a; Yang et al., 2024; Benli et al., 2025; Campos et al., 2025; Cezar et al., 2025; Costa et al., 2025; Elsherif et al., 2025; Fang et al., 2025; Lian et al., 2025; Mohamed et al., 2025; Pedrazzi et al., 2025) and heightened anxiety-like behaviors (Afshari et al., 2024; Chen S et al., 2024; Liu et al., 2024; Meng et al., 2024; Qiu et al., 2024; Reza naghdi et al., 2024; Souza et al., 2024; Sun et al., 2024; Wang et al., 2024a; Wang et al., 2024; Davoudi et al., 2025; Elsherif et al., 2025; Fang et al., 2025; Hosseini et al., 2025; Tarahomi et al., 2025). Additional frequently observed outcomes included impaired balance strength (Tarahomi et al., 2025), delayed spatial learning (Chen et al., 2024; Kurahashi et al., 2024; Sun et al., 2024; Costa et al., 2025; Tarahomi et al., 2025), reduced sensorimotor coordination and locomotor activity (Alnakhli et al., 2024; Chen et al., 2024; Lu et al., 2024; Rahdar et al., 2024; Wang et al., 2024a; Benli et al., 2025; Jiang et al., 2025), decreased social novelty preference (Meng et al., 2024; Ohnami et al., 2024; Qiu et al., 2024; Reza naghdi et al., 2024; Souza et al., 2024; Wang et al., 2024b; Benli et al., 2025; Costa et al., 2025; Davoudi et al., 2025; He et al., 2025; Lian et al., 2025; Pedrazzi et al., 2025), increased stereotypical movements (Rahdar et al., 2024; Wang et al., 2024a; Hosseini et al., 2025; Pedrazzi et al., 2025), enhanced repetitive behaviors (Chen et al., 2024; Godavarthi et al., 2024; Kurahashi et al., 2024; Liu et al., 2024; Lu et al., 2024; Meng et al., 2024; Qiu et al., 2024; Reza naghdi et al., 2024; Souza et al., 2024; Fang et al., 2025; Jiang et al., 2025; Lian et al., 2025), elevated self-grooming (Chen et al., 2024; Meng et al., 2024; Reza naghdi et al., 2024; Souza et al., 2024; Fang et al., 2025; Hosseini et al., 2025; Jiang et al., 2025; Mohamed et al., 2025; Yang et al., 2025), increased freezing response (Chu et al., 2025), hyperactivity (Cezar et al., 2025), impaired novel object recognition (Chen et al., 2024; Farbin et al., 2024; Souza et al., 2024; Sun et al., 2024), speech disorder (Tamaoki et al., 2024), abnormal auditory processing (Tamaoki et al., 2024), anxiety disorder (Afshari et al., 2024; Meng et al., 2024), cognitive impairment (King et al., 2024c; Kurahashi et al., 2024; Miao et al., 2024), social dependency, and memory impairment (Chen et al., 2024; Prince et al., 2024; Reza naghdi et al., 2024).

## Effects of valproic acid on inflammation

Inflammatory alterations were consistent across studies. Campos et al. demonstrated that 600 mg/kg i.p. VPA increased brain inflammation and altered cytokine levels (Campos et al., 2025). Another study reported elevated serum MDA and reduced GSH and catalase following 500 mg/kg VPA (Tarahomi et al., 2025). Oral VPA exposure increased serum IL-1β, IL-6, and TNF-α in male rats, whereas female rats exhibited increased brain NLRP3 levels without major changes in IL-6, TNF-α, or IL-22 (Gök Dağıdır et al., 2025). Additional studies using 600 mg/kg VPA showed increased TNF-α, IL-1β, IL-6, decreased SOD activity, and disrupted GSH/MDA balance (He et al., 2025). VPA also altered GABA/GLU balance and increased 5-HT levels (Ay et al., 2025). Other work reported disrupted SCFA metabolism, microglial activation, elevated IL-1β, IL-6, IFN-γ, TNF-α, and immune dysregulation (Palanivelu et al., 2024). Additional studies reported changes in sirtuin-1, oxidative stress markers, and dysregulation of signaling pathways including PI3K/Akt/GSK-3β, JAK2/STAT3, and PPAR-γ, together with increased ROS, NF-κB, TLR4, NOX-2, and caspase-3 (Alnakhli et al., 2024; Farbin et al., 2024; Farrag et al., 2024; Liu et al., 2024; Lu et al., 2024; Noshadian et al., 2024; Palanivelu et al., 2024; Rahdar et al., 2024; Reza naghdi et al., 2024; Saavedra-Bonilla et al., 2024; Zahedi et al., 2024; Ay et al., 2025; Gök Dağıdır et al., 2025; Tarahomi et al., 2025).

## Effects of valproic acid on histopathology

Histopathological abnormalities were widespread across rodent models. Reported findings included reduced tail length/weight ratio, altered brain weight, abnormal dendritic branching, tail malformations, reduced body weight, vascular abnormalities, neuronal degeneration in Purkinje and granule cells, thinning of myelinated fibers, loss of neurofilaments, reduced D1/D2 receptor expression, hippocampal neuronal damage, reduced brain volume, hemispheric asymmetry, altered cingulate cortex development, reduced thalamic volume and neuron count, cortical malformations, enlarged neuronal soma, reduced dendritic spine maturation, microstructural differences in the hippocampus and striatum, abnormal microglial morphology and reduced microglia density, disrupted ascending and descending auditory pathways, mitochondrial degeneration (including membrane atrophy and vacuolization), reduced synapse count, decreased serotonergic neurons in the dorsal hippocampus, increased gut permeability, reduced δ-catenin, reduced AMPA and PSD-95 levels, altered glutamatergic transmission, ultrasonic vocalization deficits, and changes in ChAT+ and PV+ cell populations (Chen et al., 2024; Ibáñez-Sandoval et al., 2024; King et al., 2024b; King et al., 2024a; Liu et al., 2024; Mansour and Kulesza, 2024; Meng et al., 2024; Neelotpol et al., 2024; Palanivelu et al., 2024; Qiu et al., 2024; Rahdar et al., 2024; Roh et al., 2024; Ay et al., 2025; Cezar et al., 2025; Chu et al., 2025; Mokhtar Tawfeek et al., 2025; Williams et al., 2025; Wu et al., 2025; Zhu et al., 2025).

## Effects of valproic acid on microbiota

Multiple studies documented gut microbiota dysbiosis following VPA exposure. Fang et al. reported decreased GMHI and increased dysbiosis index, with dominance of Bacillales, Bacillaceae, Bacillus, and Lachnospiraceae_UCG-006 in C57BL/6J mice (Fang et al., 2025). Another study showed reduced microbiota diversity in rats and identified Prevotellaceae and Peptostreptococcaceae as dominant taxa (Palanivelu et al., 2024). VPA exposure also increased intestinal permeability and disrupted microbial taxa in rats (Prince et al., 2024). In CD-1 mice receiving 250 mg/kg VPA, maternal gut alterations were observed (Li et al., 2024).

## Effects of valproic acid on gene expression

VPA induced substantial gene-expression changes across studies. Zhu et al. observed increased expression of Tbl1xr1 and Cask, and decreased Myo5a, Cacna1e, and Ap2s1, along with upregulation of numerous ASD-related genes including Grin2b, Nrxn1, Gabrb2, Mecp2, and Reln (Zhu et al., 2025). Another study reported increased mTOR phosphorylation (Ser2448) (Chu et al., 2025). A large-scale transcriptomic analysis identified alterations in 7,300 genes, including 399 ASD-risk genes (88). Additional work demonstrated decreased BDNF, increased GSK-3β (Hosseini et al., 2025), and changes in PI3K/mTOR, caspase-3, GFAP, Nestin, VEGF, NF-κB, TLR4, NOX-2, ROS, SORT1, and disruptions in ERK1/2 signaling (Alnakhli et al., 2024; Chen et al., 2024; Farrag et al., 2024; Liao et al., 2024; Reza naghdi et al., 2024; Yang et al., 2024; Campos et al., 2025; Elsherif et al., 2025; Jiang et al., 2025; Mokhtar Tawfeek et al., 2025; Wu et al., 2025). Electrophysiological studies revealed altered theta–gamma coupling, increased HG power, reduced SYP and NR2B, and increased PSD-95 (Chen B et al., 2024).

The SYRCLE risk-of-bias assessment revealed notable differences in methodological transparency across the included studies. In many cases, details regarding random sequence generation and allocation procedures were either briefly described or omitted entirely, resulting in frequent concerns regarding selection bias. Blinding practices during behavioral testing and outcome evaluation were also seldom reported, contributing to uncertainty in the assessment of performance and detection bias. The SYRCLE assessment indicated that most included studies had an overall high risk of bias, primarily because sequence generation, allocation concealment, random housing, blinding, animal-flow accounting, and litter-level experimental-unit procedures were incompletely reported. ARRIVE 2.0 reporting was generally stronger for study objectives, ethical approval, experimental procedures, and interpretation of findings, whereas inclusion and exclusion criteria, blinding, animal-care monitoring, protocol registration, and data-access procedures were frequently incomplete. Detailed study-level assessments are provided in **Supplementary Tables 1**-**4**.

## Discussion

The present review synthesized 66 eligible prenatal VPA studies published between 2024 and 2025. Across these studies, VPA consistently induced abnormalities in core behavioral domains alongside convergent molecular, inflammatory, microbiota-related, histopathological, and gene-expression alterations in rats and mice, extending and updating earlier summaries of the VPA model (Hrabovska and Salyha, 2016; El-Ansary et al., 2018; Nicolini and Fahnestock, 2018; Baker et al., 2020; Zarate-Lopez et al., 2024). Overall, recent literature suggests that prenatal VPA exposure represents a useful and widely used experimental model for investigating selected ASD-related behavioral and neurobiological phenotypes. Although behavioral similarities, partially shared mechanistic pathways, and responsiveness to selected interventions support its translational value, important methodological variability and limitations should be considered when interpreting findings.

A first point of convergence across many studies is the recurrent observation of social and repetitive behavior deficits in prenatally VPA-exposed rodents. Nevertheless, the high frequency of these reported findings should be interpreted cautiously. Behavioral outcomes may be influenced by methodological differences in species, strains, exposure timing, and behavioral paradigms, and selective emphasis on commonly assessed social endpoints may contribute to potential reporting bias across the literature. Numerous studies reported reduced sociability, impaired social interaction, or attenuated social novelty preference in offspring exposed prenatally to VPA (Afshari et al., 2024; Alnakhli et al., 2024; Chen et al., 2024; Chen et al., 2024; Farbin et al., 2024; Farrag et al., 2024; Godavarthi et al., 2024; Horata et al., 2024; Kurahashi et al., 2024; Liao et al., 2024; Liu et al., 2024; Liu et al., 2024; Lu et al., 2024; Meng et al., 2024; Miao et al., 2024; Noshadian et al., 2024; Ohnami et al., 2024; Prince et al., 2024; Qiu et al., 2024; Reza naghdi et al., 2024; Souza et al., 2024; Sun et al., 2024; Sun et al., 2024; Wang et al., 2024b; Wang et al., 2024a; Yang et al., 2024; Benli et al., 2025; Campos et al., 2025; Cezar et al., 2025; Costa et al., 2025; Elsherif et al., 2025; Fang et al., 2025; Lian et al., 2025; Mohamed et al., 2025; Pedrazzi et al., 2025; Zhu et al., 2025). These social impairments were often accompanied by reduced exploratory behavior in open-field or three-chamber tasks and diminished motivation to explore novel environments (Chen et al., 2024; Meng et al., 2024; Ohnami et al., 2024; Qiu et al., 2024; Reza naghdi et al., 2024; Souza et al., 2024; Wang et al., 2024b; Benli et al., 2025; Davoudi et al., 2025; Fang et al., 2025; He et al., 2025; Lian et al., 2025; Zhu et al., 2025). Anxiety-like behaviors were also frequently observed, including increased time spent in dark or peripheral zones, reduced center time, and heightened avoidance or freezing responses (Farbin et al., 2024; Godavarthi et al., 2024; Liao et al., 2024; Liu et al., 2024; Ohnami et al., 2024; Rahdar et al., 2024; Sun et al., 2024; Wang et al., 2024b; Wang et al., 2024a; Wang et al., 2024; Campos et al., 2025; Cezar et al., 2025; Davoudi et al., 2025; He et al., 2025; Jiang et al., 2025; Yang et al., 2025). Many studies further documented repetitive or stereotypic movements, excessive grooming, perseverative behaviors, hyperactivity, and impairments in recognition memory and spatial learning (Chen et al., 2024; Farbin et al., 2024; Godavarthi et al., 2024; Ibáñez-Sandoval et al., 2024; King et al., 2024c; King et al., 2024b; King et al., 2024a; Kurahashi et al., 2024; Lu et al., 2024; Meng et al., 2024; Palanivelu et al., 2024; Prince et al., 2024; Qiu et al., 2024; Rahdar et al., 2024; Reza naghdi et al., 2024; Saavedra-Bonilla et al., 2024; Souza et al., 2024; Sun et al., 2024; Tamaoki et al., 2024; Zahedi et al., 2024; Ay et al., 2025; Cezar et al., 2025; Chu et al., 2025; Fang et al., 2025; Gök Dağıdır et al., 2025; Hosseini et al., 2025; Jiang et al., 2025; Lian et al., 2025; Mohamed et al., 2025; Pedrazzi et al., 2025; Yang et al., 2025). Together, these findings suggest that prenatal VPA exposure reproduces several behavioral domains relevant to ASD; however, the apparent consistency of these outcomes should be interpreted cautiously in light of methodological heterogeneity and potential reporting bias across studies.

At the mechanistic level, a large proportion of the included studies highlighted neuroinflammatory and oxidative stress pathways as central components of the VPA phenotype. Increases in TNF-α, IL-1β, IL-6, IFN-γ, malondialdehyde (MDA), reactive oxygen species (ROS), and related pro-inflammatory markers were repeatedly reported following VPA administration (Farbin et al., 2024; Farrag et al., 2024; Liu et al., 2024; Lu et al., 2024; Noshadian et al., 2024; Palanivelu et al., 2024; Rahdar et al., 2024; Reza naghdi et al., 2024; Saavedra-Bonilla et al., 2024; Ay et al., 2025; Campos et al., 2025; He et al., 2025; Tarahomi et al., 2025). In parallel, reductions in antioxidant defenses such as glutathione (GSH), glutathione peroxidase (GPX), superoxide dismutase (SOD), and catalase were observed, suggesting a shift toward a pro-oxidant state (Farbin et al., 2024; Ay et al., 2025; Tarahomi et al., 2025). Several studies described activation of the NLRP3 inflammasome and upregulation of NF-κB, TLR4, NOX-2, and caspase-3, together with enhanced microglial and astrocytic reactivity and altered microglial morphology (Alnakhli et al., 2024; Farbin et al., 2024; Farrag et al., 2024; Liao et al., 2024; Mansour and Kulesza, 2024; Reza naghdi et al., 2024; Yang et al., 2024; Ay et al., 2025; Campos et al., 2025; Jiang et al., 2025; Williams et al., 2025). These findings suggest that prenatal VPA exposure can alter fetal neuroimmune regulation and promote inflammatory signaling. Such changes resemble aspects of neuroimmune dysregulation reported across several ASD experimental models, including maternal immune activation (MIA), genetic models targeting synaptic genes such as Shank3 and Cntnap2, and other environmentally induced paradigms. Nevertheless, these parallels should be interpreted cautiously and should not be viewed as evidence of direct causal correspondence with human ASD (Lammert and Lukens, 2019; Han et al., 2021; Cerilli et al., 2024; Li, 2024).

Histopathological and neuroanatomical findings provide complementary structural evidence for the impact of VPA on brain development. Across multiple studies, VPA-exposed offspring showed reductions in brain weight and brain/body ratios, decreased tail length, and overt malformations such as tail deformities and altered growth trajectories (Cezar et al., 2025; Chu et al., 2025; Zhu et al., 2025). At the cellular level, investigators reported cortical malformations, impaired dendritic branching, reduced dendritic spine maturation, Purkinje and granule cell degeneration, thinning of myelinated fibers, loss of neurofilaments, and hippocampal neuronal damage (Chen et al., 2024; Ibáñez-Sandoval et al., 2024; King et al., 2024b; King et al., 2024a; Liu et al., 2024; Mansour and Kulesza, 2024; Meng et al., 2024; Qiu et al., 2024; Rahdar et al., 2024; Zahedi et al., 2024; Ay et al., 2025; Cezar et al., 2025; He et al., 2025; Williams et al., 2025; Zhu et al., 2025). Region-specific volumetric abnormalities were described in structures such as the anterior cingulate cortex, hippocampus, and cerebellar lobules, often with sex-dependent patterns (Finszter et al., 2024; Horata et al., 2024; Ibáñez-Sandoval et al., 2024; King et al., 2024c; King et al., 2024b; King et al., 2024a; Lian et al., 2025). Several studies also documented altered serotonergic and dopaminergic neuron populations, changes in auditory pathway projections, and abnormalities in brainstem connectivity and auditory brainstem responses (Finszter et al., 2024; Horata et al., 2024; King et al., 2024a; Tamaoki et al., 2024). These structural and circuit-level alterations align with human ASD findings implicating cortico-striatal, cerebellar, and limbic circuitry, and suggest that VPA models capture key aspects of ASD-relevant neuroanatomical vulnerability.

The emerging literature on the microbiota–gut–brain axis in VPA models adds an important systems-level dimension. VPA-exposed rodents frequently exhibited reduced gut microbial diversity, shifts in taxonomic composition (e.g. dominance of Bacillales, Bacillaceae, Prevotellaceae, Peptostreptococcaceae, Lachnospiraceae_UCG-006), and increased intestinal permeability (Prince et al., 2024; Ay et al., 2025; Fang et al., 2025). Maternal gut dysbiosis and pathological alterations in maternal intestinal structure and motility were also reported in association with prenatal VPA exposure (Yang et al., 2024; Ay et al., 2025; Wu et al., 2025). These changes were often paralleled by elevated systemic and central inflammatory markers and disturbances in short-chain fatty acid metabolism (Liu et al., 2024; Yang et al., 2024; Ay et al., 2025; Fang et al., 2025). Although causality cannot be fully established from the available studies, the convergence of microbiota dysbiosis, barrier dysfunction, and neurobehavioral alterations supports a mechanistic contribution of gut–brain signaling pathways to ASD-like phenotypes in the VPA model.

On the molecular level, gene-expression and signaling studies revealed extensive transcriptional remodeling following prenatal VPA exposure. Zhu et al. reported upregulation of genes such as Tbl1xr1, Cask, Grin2b, Nrxn1, Taok1, and others, along with downregulation of Myo5a, Cacna1e, and Ap2s1, implicating multiple ASD-relevant synaptic and neurodevelopmental genes in the VPA phenotype (Zhu et al., 2025). Another study documented altered expression in approximately 7,300 genes-including 399 ASD risk genes and 258 non-ASD genes-highlighting the breadth of VPA effects on the fetal transcriptome (Li et al., 2024). Increased mTOR phosphorylation, changes in GSK-3β activity, and reduced BDNF levels were also reported, consistent with dysregulated PI3K/Akt/mTOR and neuroplasticity pathways (Alnakhli et al., 2024; Chen B et al., 2024; Farrag et al., 2024; Liao et al., 2024; Mansour and Kulesza, 2024; Reza naghdi et al., 2024; Yang et al., 2024; Campos et al., 2025; Chu et al., 2025; Elsherif et al., 2025; Hosseini et al., 2025; Jiang et al., 2025; Williams et al., 2025). Alongside these, disruptions in ERK1/2, JAK2/STAT3, PPAR-γ, Wnt/β-catenin, and serotonin and dopamine receptor signaling were described, as well as changes in high-frequency gamma power and theta–gamma coupling in hippocampal networks (Hall et al., 2002; Chenn, 2008; Jung et al., 2008; Sernagor, 2010; Alnakhli et al., 2024; Chen B et al., 2024; Farrag et al., 2024; Liao et al., 2024; Li et al., 2024; Mansour and Kulesza, 2024; Reza naghdi et al., 2024; Yang et al., 2024; Campos et al., 2025; Chu et al., 2025; Elsherif et al., 2025; Hosseini et al., 2025; Jiang et al., 2025; Williams et al., 2025). Collectively, these findings support the view that VPA acts as a broad epigenetic and signaling modulator that converges on pathways critical for synaptic development, excitatory–inhibitory balance, and circuit formation-key mechanisms also implicated in human ASD.

Despite these strengths, several important limitations of both the prenatal VPA model and the included studies should be acknowledged. Experimental parameters varied considerably, with VPA doses ranging from 150 to 800 mg/kg, routes of administration including intraperitoneal, subcutaneous, and oral delivery, and gestational exposure windows ranging from embryonic day 10 to E14, although E12.5 was the predominant exposure time point, although gestational day 12.5 was most frequently used (Afshari et al., 2024; Chen et al., 2024; Finszter et al., 2024; Horata et al., 2024; Ibáñez-Sandoval et al., 2024; King et al., 2024c; King et al., 2024b; King et al., 2024a; Kurahashi et al., 2024; Lu et al., 2024; Prince et al., 2024; Yang et al., 2024; Ay et al., 2025; Campos et al., 2025; Cezar et al., 2025; Chu et al., 2025; Fang et al., 2025; Lian et al., 2025; Tarahomi et al., 2025; Wu et al., 2025; Zhu et al., 2025). Such methodological heterogeneity complicates direct comparisons across studies and may partially account for inconsistencies in reported findings. Importantly, recent pharmacokinetic and protocol-oriented investigations demonstrated that route of administration and dosage substantially influence VPA exposure profiles and fetal outcomes. Distinct administration routes generate differences in absorption, peak concentrations, systemic exposure, and elimination patterns, while dose selection and exposure timing may affect embryotoxicity and behavioral expression (Tartaglione et al., 2019; Morel et al., 2024). Oral administration has also been associated with greater variability and higher fetal toxicity compared with intraperitoneal administration (Morel et al., 2024). Experimental protocols using single-dose exposure around gestational day 12.5 may create a brief but developmentally critical window capable of influencing neurogenesis, cortical organization, and subsequent behavioral outcomes (Tartaglione et al., 2019; Dorsey et al., 2024). Furthermore, several investigations did not clearly report methodological procedures such as randomization, blinding, litter allocation, or sample size determination, increasing the risk of bias and limiting reproducibility.

From a translational perspective, it is also crucial to emphasize that prenatal VPA exposure in rodents reproduces ASD-like features rather than replicating the full clinical and etiological complexity of human ASD. ASD in humans typically arises from polygenic and heterogeneous environmental contributions, whereas prenatal VPA administration is a relatively acute and high-impact insult. Although clinical evidence links *in utero* VPA exposure with increased risk of neurodevelopmental problems and ASD-related features (Zohny et al., 2023; Zarate-Lopez et al., 2024), findings from rodent VPA models should not be interpreted as direct proof that VPA universally causes ASD in humans. Instead, they should be viewed as experimental tools that capture specific dimensions of ASD-related pathology (particularly social behavior, repetitive behavior, neuroinflammation, synaptic dysfunction, and gut–brain alterations) under controlled conditions. These considerations can be further examined within the framework of classical animal-model validity criteria, including face, construct, and predictive validity, which provide a structured basis for assessing the translational relevance of experimental ASD models.

### Validity of the prenatal VPA model of ASD

The translational relevance of prenatal VPA exposure can be evaluated according to classical animal-model validity criteria, including face, construct, and predictive validity (Nicolini and Fahnestock, 2018; Mehra et al., 2022; Zarate-Lopez et al., 2024). Face validity refers to the extent to which an animal model reproduces behavioral characteristics observed in humans. Across numerous studies, prenatally VPA-exposed rodents frequently exhibit social interaction deficits, impaired communication, repetitive behaviors, anxiety-like traits, and sensory abnormalities resembling ASD-related phenotypes, although variability exists across experimental protocols (Nicolini and Fahnestock, 2018; Mehra et al., 2022; Ukezono et al., 2024; Zarate-Lopez et al., 2024). Construct validity reflects similarities in etiological and mechanistic pathways. Prenatal VPA exposure is supported by epidemiological evidence linking gestational VPA administration with increased ASD risk (Zohny et al., 2023; Zarate-Lopez et al., 2024) and reproduces several biological pathways implicated in ASD pathophysiology, including HDAC inhibition, epigenetic dysregulation, altered excitatory–inhibitory balance, neuroinflammation, abnormal neurodevelopmental signaling pathways, and widespread fetal transcriptomic alterations affecting ASD-related genes (Nicolini and Fahnestock, 2018; Mehra et al., 2022; Dorsey et al., 2024; Zarate-Lopez et al., 2024). Predictive validity concerns the ability of a model to respond to interventions in a manner relevant to human pathology. Although several pharmacological, behavioral, and microbiota-targeted interventions have shown partial reversal of ASD-like phenotypes in VPA-exposed rodents, evidence supporting predictive validity remains comparatively limited (Nicolini and Fahnestock, 2018; Mehra et al., 2022). Nevertheless, despite these strengths, the prenatal VPA model does not fully reproduce the etiological heterogeneity and clinical complexity of human ASD and should therefore be considered a model of specific behavioral and neurobiological dimensions relevant to ASD rather than a complete representation of the disorder.

Collectively, available evidence supports an integrative pathophysiological framework in which prenatal VPA exposure initially induces epigenetic and transcriptomic dysregulation through HDAC inhibition (Hall et al., 2002; Chenn, 2008; Jung et al., 2008; Sernagor, 2010; Nicolini and Fahnestock, 2018; Dorsey et al., 2024). These early molecular events may subsequently disrupt critical neurodevelopmental processes, including neuronal proliferation, differentiation, migration, cortical lamination, cortical patterning, and synaptic maturation. Alterations in developmental signaling pathways such as Wnt/β-catenin, PI3K/Akt/mTOR, ERK, and GSK-3β may further disturb excitatory–inhibitory balance and impair normal circuit assembly (Hall et al., 2002; Chenn, 2008; Jung et al., 2008; Sernagor, 2010; Alnakhli et al., 2024; Chen B et al., 2024; Farrag et al., 2024; Liao et al., 2024; Mansour and Kulesza, 2024; Reza naghdi et al., 2024; Yang et al., 2024; Campos et al., 2025; Chu et al., 2025; Elsherif et al., 2025; Hosseini et al., 2025; Jiang et al., 2025; Williams et al., 2025). In parallel, prenatal VPA exposure is associated with immune dysregulation characterized by microglial activation, increased inflammatory mediators, oxidative stress, and altered cytokine signaling (Farbin et al., 2024; Farrag et al., 2024; Li et al., 2024; Reza naghdi et al., 2024; Yang et al., 2024; Ay et al., 2025; Campos et al., 2025). Gut microbiota disturbances and impaired intestinal barrier function may further contribute to systemic inflammatory responses and gut–brain communication abnormalities (Prince et al., 2024; Yang et al., 2024; Ay et al., 2025; Fang et al., 2025; Wu et al., 2025). Emerging evidence additionally suggests that alterations in neurotransmitter systems may represent an important mechanistic bridge linking early VPA exposure to later behavioral abnormalities. Prenatal VPA exposure has been associated with changes in GABAergic signaling, including altered expression of glutamate decarboxylase enzymes, vesicular GABA transporters, GABA receptor subunits, and parvalbumin-related pathways, accompanied by abnormal neurite branching and disturbances in cortical excitatory–inhibitory balance (Adıgüzel et al., 2025; Mihalj et al., 2025). Serotonergic mechanisms may also contribute substantially, as alterations in serotonin signaling and dysregulated 5-HT7 receptor expression have been linked to hippocampal hyperexcitability, synaptic dysfunction, and ASD-like behavioral abnormalities in VPA-exposed offspring (Adiguzel et al., 2024; Rahdar et al., 2024). Together, these convergent molecular, immune, neurotransmitter-related, and neurodevelopmental alterations may interfere with circuit maturation, synaptic connectivity, and network-level organization, ultimately contributing to ASD-like behavioral phenotypes.

In summary, the studies reviewed here suggest that prenatal VPA exposure remains a widely used and informative experimental model for investigating selected ASD-related behavioral and neurobiological mechanisms. Recent work has expanded this model beyond classical behavioral testing to incorporate neuroimmune profiling, advanced histopathology, multi-omics transcriptomics, microbiome sequencing, and circuit-level electrophysiology (Chen B et al., 2024; Farbin et al., 2024; Finszter et al., 2024; Ibáñez-Sandoval et al., 2024; King et al., 2024c; King et al., 2024b; King et al., 2024a; Li et al., 2024; Liu et al., 2024; Yang et al., 2024; Yu et al., 2024; Ay et al., 2025; Campos et al., 2025; Chu et al., 2025; Fang et al., 2025; Lian et al., 2025; Zhu et al., 2025). Future research will benefit from greater harmonization of experimental protocols (dose, timing, strain, sex), rigorous implementation and reporting of bias-reducing strategies, systematic integration of behavioral and biological readouts, and direct comparison of VPA models with genetic and other environmental ASD models. Such efforts may improve understanding of which dimensions of ASD pathophysiology are most appropriately represented by the prenatal VPA model.

## Conclusion

Recent rodent research using prenatal VPA exposure continues to reveal consistent disruptions in behavioral, molecular, and neurobiological processes relevant to ASD. Across studies conducted over the past two years, VPA-exposed offspring commonly showed alterations in social behavior, repetitive actions, neuroinflammatory responses, gene-expression patterns, gut microbiota composition, and brain structure. These convergent findings highlight the utility of the VPA model for probing specific pathways implicated in ASD. Even so, the considerable variation in species, strains, doses, and gestational timing across studies, together with incomplete methodological reporting, limits cross-study comparability and may contribute to variability in behavioral and biological outcomes. Thus, the prenatal VPA model should be viewed as reproducing selected ASD-like phenotypes and neurobiological dimensions rather than fully recapitulating the etiological heterogeneity and clinical complexity of ASD in humans.

## Data Availability

The original contributions presented in the study are included in the article/**Supplementary Material**, further inquiries can be directed to the corresponding author.
