# Supplementary material for: Prenatal valproic acid exposure in rodent models of autism: a systematic review of neurobehavioral physiological alterations
Source: Front Physiol. 2026 Sep 7;17:1860596. doi: 10.3389/fphys.2026.1860596 (PMC13590398; doi:10.3389/fphys.2026.1860596)
Supplement: Supplementary file 1 [file Table1.docx]

**SUPPLEMENTARY FILE**

**Prenatal Valproic Acid Exposure in Rodent Models of Autism:
A Systematic Review of Neurobehavioral and Physiological Alterations**

*Final methodological quality, risk-of-bias, and reporting-completeness assessment*

**Methods.** Risk of bias was assessed with the 10-domain SYRCLE tool. ARRIVE 2.0 was used separately to evaluate reporting completeness. Low risk/adequate reporting was assigned only when the relevant procedure was explicitly described. Unreported methods were classified conservatively as unclear risk or not reported. Potential litter-level pseudoreplication was considered under 'Other bias'.

**Supplementary Table S1. Study-level SYRCLE risk-of-bias judgments**

| **Ref** | **Study** | **D1** | **D2** | **D3** | **D4** | **D5** | **D6** | **D7** | **D8** | **D9** | **D10** | **Overall** |
| --- | --- | --- | --- | --- | --- | --- | --- | --- | --- | --- | --- | --- |
| 19 | Mechanistic insights into retinoic-acid treatment for autism in the improvement of social behavior: Evidence from a multi omics study in rats | U | U | U | U | U | U | L | U | U | H | H |
| 21 | Promising therapeutic effects of high-frequency repetitive transcranial magnetic stimulation (HF-rTMS) in addressing autism spectrum disorder induced by valproic acid | U | U | U | U | U | U | U | U | U | H | H |
| 22 | Perindopril Ameliorates Sodium Valproate-Induced Rat Model of Autism | U | U | U | U | U | U | U | U | U | H | H |
| 23 | Genetic machinery which accompanies metaplasticity operates differentially in experimental model of autism | U | U | U | U | U | U | L | U | U | H | H |
| 24 | Animal Model of Autism Induced by Valproic Acid Combined with Maternal Deprivation: Sex-Specific Effects on Inflammation and Oxidative Stress | U | U | U | U | U | U | U | U | U | H | H |
| 25 | Prenatal Valproic Acid Induces Autistic‐Like Behaviors in Rats via Dopaminergic Modulation in Nigrostriatal and Mesocorticolimbic Pathways | U | U | U | U | U | U | U | U | U | H | H |
| 26 | VPA-induced autism impairs memory ability through disturbing neural oscillatory patterns in offspring rats | U | U | U | U | U | U | U | U | U | H | H |
| 27 | Chinese acupuncture: A potential treatment for autism rat model via improving synaptic function | U | U | U | U | U | L | U | U | U | H | H |
| 28 | Effects of Cannabidiol Isolated or in Association With Risperidone in an Animal Model of Autism | U | U | U | U | U | U | U | U | U | H | H |
| 29 | The potential ameliorative effect of mesenchymal stem cells-derived exosomes on cerebellar histopathology and their modifying role on PI3k-mTOR signaling in rat model of autism spectrum disorder | U | U | U | U | U | U | U | U | U | H | H |
| 30 | Integrating 16S rRNA Gene Sequencing and Metabolomics Analysis to Reveal the Mechanism of L-Proline in Preventing Autism-like Behavior in Mice | U | U | U | U | U | U | L | U | U | H | H |
| 31 | Neuroprotective effects of Apigenin on prenatal Valproic acid-induced autism spectrum disorder in rats | U | U | U | U | U | U | U | U | U | H | H |
| 32 | Comparative effect of atorvastatin and risperidone on modulation of TLR4/NF-κB/NOX-2 in a rat model of valproic acid-induced autism | U | U | U | U | U | L | L | L | U | H | H |
| 33 | Embryonic exposure to environmental factors drives transmitter switching in the neonatal mouse cortex causing autistic-like adult behavior | U | U | U | U | U | U | U | U | U | H | H |
| 35 | Autistic-like behaviour and changes in thalamic cell numbers a rat model of valproic acid-induced autism; A behavioural and stereological study | U | U | U | U | U | U | U | L | L | H | H |
| 36 | Autism spectrum disorder-like behaviors induced by hyper-glutamatergic NMDA receptor signaling through hypo-serotonergic 5-HT1A receptor signaling | U | U | U | U | U | U | U | U | U | H | H |
| 37 | Developmental Trajectory of Autistic‐Like Behaviors in a Prenatal Valproic Acid Rat Model of Autism | U | U | U | U | U | U | U | U | U | H | H |
| 38 | Sortilin is associated with progranulin deficiency and autism‐like behaviors in valproic acid‐induced autism rats | U | U | U | U | U | U | U | U | U | H | H |
| 39 | Clostridium butyricum regulates intestinal barrier function via trek1 to improve behavioral abnormalities in mice with autism spectrum disorder | U | U | U | U | U | L | U | U | U | H | H |
| 40 | Administration of Atosiban, an oxytocin receptor antagonist, ameliorates autistic-like behaviors in a female rat model of valproic acid-induced autism | U | U | U | U | U | U | U | U | U | H | H |
| 41 | Oral edaravone ameliorates behavioral deficits and pathologies in a valproic acid-induced rat model of autism spectrum disorder | U | U | U | U | U | U | U | U | U | H | H |
| 42 | Transient CSF1R inhibition ameliorates behavioral deficits in Cntnap2 knockout and valproic acid-exposed mouse models of autism | U | U | U | U | U | U | U | U | U | H | H |
| 43 | Biomimetic nanoparticles with enhanced rapamycin delivery for autism spectrum disorder treatment | U | U | U | U | U | U | U | U | U | H | H |
| 44 | Sigma-1 receptor modulation by fluvoxamine ameliorates valproic acid-induced autistic behavior in rats: Involvement of chronic ER stress modulation, enhanced autophagy and M1/M2 microglia polarization | U | U | U | U | U | U | L | U | U | H | H |
| 45 | Benefits of bone marrow mesenchymal stem cells compared to their conditioned medium in valproic acid-induced autism in rats | U | U | U | U | U | U | U | U | U | H | H |
| 46 | Brain region-specific neural activation by low-dose opioid promotes social behavior | U | U | U | U | U | U | U | U | U | H | H |
| 47 | Acute cannabidiol treatment reverses behavioral impairments induced by embryonic valproic acid exposure in male mice | U | U | U | U | U | U | U | U | U | H | H |
| 48 | Prebiotic diet normalizes aberrant immune and behavioral phenotypes in a mouse model of autism spectrum disorder | U | U | U | U | U | U | U | U | U | H | H |
| 50 | The neuroprotective effect of Diosgenin in the rat Valproic acid model of autism | U | U | U | U | U | U | U | U | U | H | H |
| 51 | An alpha 5‐GABA A receptor positive allosteric modulator attenuates social and cognitive deficits without changing dopamine system hyperactivity in rats exposed to valproic acid in utero | U | U | U | U | U | U | U | U | U | H | H |
| 52 | Human umbilical cord-derived mesenchymal stem cells alleviate valproate-induced immune stress and social deficiency in rats | U | U | U | U | U | U | U | U | U | H | H |
| 53 | Adult neurogenesis in the ventral hippocampus decreased among animal models of neurodevelopmental disorders | U | U | U | U | U | U | U | U | U | H | H |
| 54 | Restoration of nNOS Expression Rescues Autistic-Like Phenotypes Through Normalization of AMPA Receptor-Mediated Neurotransmission | U | U | U | U | U | U | U | U | U | H | H |
| 55 | Low-frequency RTMS attenuates social impairment in the VPA-induced mouse model | U | U | U | U | U | U | U | U | U | H | H |
| 56 | Supplementation with stigma maydis polysaccharide attenuates autism‐like behaviors and improves gut function in valproic acid‐induced autism model male rats | U | U | U | U | U | U | U | U | U | H | H |
| 57 | The Impact of Astroglia Kir4.1 Channel Dysfunction on Neuronal Activity and Autism‐Related Behavioral Abnormalities | U | U | U | U | U | U | U | U | U | H | H |
| 58 | BDNF and GSK-3beta expression changes underlie the beneficial effects of crocin on behavioral alterations in a rat model of autism induced by prenatal valproic acid administration | U | U | U | U | U | U | U | U | U | H | H |
| 59 | Microglia inversely regulate the level of perineuronal nets with the treatment of lipopolysaccharide and valproic acid | U | U | U | U | U | U | U | U | U | U | U |
| 60 | Vitamin C and Gallic Acid Ameliorate Motor Dysfunction, Cognitive Deficits, and Brain Oxidative Stress | U | U | U | U | U | U | U | U | U | H | H |
| 61 | Taprenepag restores maternal–fetal interface homeostasis for the treatment of neurodevelopmental disorders | U | U | U | U | U | U | U | U | U | H | H |
| 62 | Lipopolysaccharide induces neuroinflammation in a valproic acid male model of autism | U | U | U | U | U | U | U | U | U | L | U |
| 63 | Reversal of electrophysiological and behavioral deficits mediated by 5-HT7 receptor upregulation following LP-211 treatment in an autistic-like rat model induced by prenatal valproic acid exposure | U | U | U | U | U | U | U | U | U | H | H |
| 64 | NEAT1 Promotes Valproic Acid-Induced Autism Spectrum Disorder by Recruiting YY1 to Regulate UBE3A Transcription | U | U | U | U | U | L | U | U | U | H | H |
| 65 | A critical period for paired-housing-dependent autistic-like behaviors attenuation | U | U | U | U | U | U | U | U | U | H | H |
| 66 | Soluble epoxide hydrolase deletion rescues behavioral and synaptic deficits by AMPK-mTOR pathway in autism animals | U | U | U | U | U | U | U | U | U | H | H |
| 67 | Pairing tones with vagus nerve stimulation improves brain stem responses to speech in the valproic acid model of autism | U | U | U | U | U | U | L | U | U | H | H |
| 68 | Adolescent treadmill exercise enhances hippocampal brain-derived neurotrophic factor (BDNF) expression and improves cognition in autism-modeled rats | U | U | U | U | U | U | U | U | U | H | H |
| 69 | tVNS alters inflammatory response in adult VPA ‐induced mouse model of autism: evidence for sexual dimorphism | U | U | U | U | U | U | U | U | U | H | H |
| 71 | Increased Serotonin Levels and Unchanged Glutamate and GABA Levels in Thalamic Microdialysates Despite Reduced Cell Numbers in a Valproic Acid-Induced Autism Model | U | U | U | U | U | L | U | U | U | H | H |
| 72 | Investigating brain–gut microbiota dynamics and inflammatory processes in an autistic-like rat model using MRI biomarkers during childhood and adolescence | U | U | U | U | U | U | U | U | U | H | H |
| 73 | Spontaneous Calcium Transients Recorded from Striatal Astrocytes in a Preclinical Model of Autism | U | U | U | U | U | U | U | U | U | H | H |
| 74 | Acetyl-l-carnitine alleviates valproate-induced autism-like behaviors through attenuation of hippocampal mitochondrial dysregulation | U | U | U | U | U | U | U | L | U | H | H |
| 75 | Striatal Interneuron Imbalance in a Valproic Acid-Induced Model of Autism in Rodents | U | U | U | U | U | U | U | U | U | H | H |
| 76 | Altered prefrontal and cerebellar parvalbumin neuron counts are associated with cognitive changes in male rats | U | U | U | U | U | U | U | U | U | L | U |
| 77 | Region-Specific Brain Volume Changes Emerge in Adolescence in the Valproic Acid Model of Autism and Parallel Human Findings | U | U | U | U | U | U | U | U | U | H | H |
| 78 | Obliteration of a glycinergic projection to the medial geniculate in an animal model of autism | U | U | U | U | U | U | U | U | U | H | H |
| 79 | Construction of an animal model of autism based on interaction between cerebellar histological, immunohistochemical, and biochemical changes in adult male albino rat | U | U | U | U | U | U | U | U | U | H | H |
| 80 | Pharmacological intervention of behavioural traits and brain histopathology of prenatal valproic acid-induced mouse model of autism | U | U | U | U | U | U | U | U | U | H | H |
| 81 | Prenatal exposure to valproic acid reduces synaptic δ-catenin levels and disrupts ultrasonic vocalization in neonates | U | U | U | U | U | U | U | U | U | H | H |
| 82 | Prenatal exposure to valproic acid induces sex-specific alterations in rat cortical and hippocampal neuronal structure and function in vitro | U | U | U | U | U | U | U | U | L | H | H |
| 83 | Salidroside ameliorates neuroinflammation in autistic rats by inhibiting NLRP3/Caspase-1/GSDMD signal pathway | U | U | U | U | U | U | U | U | U | H | H |
| 84 | Gastrointestinal problems in a valproic acid-induced rat model of autism: From maternal intestinal health to offspring intestinal function | U | U | U | U | U | U | U | U | U | H | H |
| 85 | Continuous exercise training rescues hippocampal long-term potentiation in the VPA rat model of Autism: Uncovering sex-specific effects | U | U | U | U | U | U | U | U | U | H | H |
| 86 | Aerobic exercise improves cognitive flexibility and modulates regional volume changes in a rat model of autism | U | U | U | U | U | U | U | U | U | H | H |
| 87 | KCNH5 deletion increases autism susceptibility by regulating neuronal growth through Akt/mTOR signaling pathway | U | U | U | U | U | U | U | U | U | H | H |
| 88 | Gestational VPA exposure reduces the density of juxtapositions between TH+ axons and calretinin or calbindin expressing cells | U | U | U | U | U | L | L | U | U | U | U |

D1 sequence generation; D2 baseline characteristics; D3 allocation concealment; D4 random housing; D5 blinding of caregivers/investigators; D6 random outcome assessment; D7 blinding of outcome assessor; D8 incomplete outcome data; D9 selective reporting; D10 other bias. L, low risk; U, unclear risk; H, high risk.

**Supplementary Table S2. Key SYRCLE rationale and page-level evidence**

| **Ref** | **Study** | **Overall** | **Principal rationale** | **PDF page(s)** |
| --- | --- | --- | --- | --- |
| 19 | Mechanistic insights into retinoic-acid treatment for autism in the improvement of social behavior: Evidence from a multi omics study in rats | H | Offspring were analyzed without clear statistical control for litter clustering, creating potential pseudoreplication bias. | 2 |
| 21 | Promising therapeutic effects of high-frequency repetitive transcranial magnetic stimulation (HF-rTMS) in addressing autism spectrum disorder induced by valproic acid | H | Dam/litter allocation, pups selected per litter, or clustering of littermates was not adequately addressed, creating potential unit-of-analysis bias. | 4 |
| 22 | Perindopril Ameliorates Sodium Valproate-Induced Rat Model of Autism | H | Dam/litter allocation, pups selected per litter, or clustering of littermates was not adequately addressed, creating potential unit-of-analysis bias. | 2 |
| 23 | Genetic machinery which accompanies metaplasticity operates differentially in experimental model of autism | H | Prenatally exposed offspring were analyzed without clear dam/litter allocation or litter-level control. | - |
| 24 | Animal Model of Autism Induced by Valproic Acid Combined with Maternal Deprivation: Sex-Specific Effects on Inflammation and Oxidative Stress | H | Offspring were analyzed without clear statistical control for litter clustering, creating potential pseudoreplication bias. | 2 |
| 25 | Prenatal Valproic Acid Induces Autistic‐Like Behaviors in Rats via Dopaminergic Modulation in Nigrostriatal and Mesocorticolimbic Pathways | H | Offspring were analyzed without clear statistical control for litter clustering, creating potential pseudoreplication bias. | 2 |
| 26 | VPA-induced autism impairs memory ability through disturbing neural oscillatory patterns in offspring rats | H | Offspring were analyzed without clear statistical control for litter clustering, creating potential pseudoreplication bias. | 2 |
| 27 | Chinese acupuncture: A potential treatment for autism rat model via improving synaptic function | H | Offspring were analyzed without clear statistical control for litter clustering, creating potential pseudoreplication bias. | 1 |
| 28 | Effects of Cannabidiol Isolated or in Association With Risperidone in an Animal Model of Autism | H | Offspring were analyzed without clear statistical control for litter clustering, creating potential pseudoreplication bias. | 4 |
| 29 | The potential ameliorative effect of mesenchymal stem cells-derived exosomes on cerebellar histopathology and their modifying role on PI3k-mTOR signaling in rat model of autism spectrum disorder | H | Offspring were analyzed without clear statistical control for litter clustering, creating potential pseudoreplication bias. | 1 |
| 30 | Integrating 16S rRNA Gene Sequencing and Metabolomics Analysis to Reveal the Mechanism of L-Proline in Preventing Autism-like Behavior in Mice | H | Dam/litter allocation, pups selected per litter, or clustering of littermates was not adequately addressed, creating potential unit-of-analysis bias. | 6 |
| 31 | Neuroprotective effects of Apigenin on prenatal Valproic acid-induced autism spectrum disorder in rats | H | Offspring were analyzed without clear statistical control for litter clustering, creating potential pseudoreplication bias. | 2 |
| 32 | Comparative effect of atorvastatin and risperidone on modulation of TLR4/NF-κB/NOX-2 in a rat model of valproic acid-induced autism | H | Dam/litter allocation, pups selected per litter, or clustering of littermates was not adequately addressed, creating potential unit-of-analysis bias. | 2 |
| 33 | Embryonic exposure to environmental factors drives transmitter switching in the neonatal mouse cortex causing autistic-like adult behavior | H | Offspring were analyzed without clear statistical control for litter clustering, creating potential pseudoreplication bias. | 1 |
| 35 | Autistic-like behaviour and changes in thalamic cell numbers a rat model of valproic acid-induced autism; A behavioural and stereological study | H | Offspring were analyzed without clear statistical control for litter clustering, creating potential pseudoreplication bias. | 1 |
| 36 | Autism spectrum disorder-like behaviors induced by hyper-glutamatergic NMDA receptor signaling through hypo-serotonergic 5-HT1A receptor signaling | H | Dam/litter allocation, pups selected per litter, or clustering of littermates was not adequately addressed, creating potential unit-of-analysis bias. | NR |
| 37 | Developmental Trajectory of Autistic‐Like Behaviors in a Prenatal Valproic Acid Rat Model of Autism | H | Offspring were analyzed without clear statistical control for litter clustering, creating potential pseudoreplication bias. | 3 |
| 38 | Sortilin is associated with progranulin deficiency and autism‐like behaviors in valproic acid‐induced autism rats | H | Prenatally exposed offspring were analyzed without clear dam/litter allocation or litter-level control. | - |
| 39 | Clostridium butyricum regulates intestinal barrier function via trek1 to improve behavioral abnormalities in mice with autism spectrum disorder | H | Offspring were analyzed without clear statistical control for litter clustering, creating potential pseudoreplication bias. | 2 |
| 40 | Administration of Atosiban, an oxytocin receptor antagonist, ameliorates autistic-like behaviors in a female rat model of valproic acid-induced autism | H | Offspring were analyzed without clear statistical control for litter clustering, creating potential pseudoreplication bias. | 2 |
| 41 | Oral edaravone ameliorates behavioral deficits and pathologies in a valproic acid-induced rat model of autism spectrum disorder | H | Offspring were analyzed without clear statistical control for litter clustering, creating potential pseudoreplication bias. | 2 |
| 42 | Transient CSF1R inhibition ameliorates behavioral deficits in Cntnap2 knockout and valproic acid-exposed mouse models of autism | H | Offspring were analyzed without clear statistical control for litter clustering, creating potential pseudoreplication bias. | 3 |
| 43 | Biomimetic nanoparticles with enhanced rapamycin delivery for autism spectrum disorder treatment | H | Dam/litter allocation, pups selected per litter, or clustering of littermates was not adequately addressed, creating potential unit-of-analysis bias. | 1 |
| 44 | Sigma-1 receptor modulation by fluvoxamine ameliorates valproic acid-induced autistic behavior in rats: Involvement of chronic ER stress modulation, enhanced autophagy and M1/M2 microglia polarization | H | Offspring were analyzed without clear statistical control for litter clustering, creating potential pseudoreplication bias. | 1 |
| 45 | Benefits of bone marrow mesenchymal stem cells compared to their conditioned medium in valproic acid-induced autism in rats | H | Offspring were analyzed without clear statistical control for litter clustering, creating potential pseudoreplication bias. | 2 |
| 46 | Brain region-specific neural activation by low-dose opioid promotes social behavior | H | Dam/litter allocation, pups selected per litter, or clustering of littermates was not adequately addressed, creating potential unit-of-analysis bias. | 13 |
| 47 | Acute cannabidiol treatment reverses behavioral impairments induced by embryonic valproic acid exposure in male mice | H | Prenatally exposed offspring were analyzed without clear dam/litter allocation or litter-level control. | - |
| 48 | Prebiotic diet normalizes aberrant immune and behavioral phenotypes in a mouse model of autism spectrum disorder | H | Prenatally exposed offspring were analyzed without clear dam/litter allocation or litter-level control. | - |
| 50 | The neuroprotective effect of Diosgenin in the rat Valproic acid model of autism | H | Prenatally exposed offspring were analyzed without clear dam/litter allocation or litter-level control. | - |
| 51 | An alpha 5‐GABA A receptor positive allosteric modulator attenuates social and cognitive deficits without changing dopamine system hyperactivity in rats exposed to valproic acid in utero | H | Offspring were analyzed without clear statistical control for litter clustering, creating potential pseudoreplication bias. | 4 |
| 52 | Human umbilical cord-derived mesenchymal stem cells alleviate valproate-induced immune stress and social deficiency in rats | H | Dam/litter allocation, pups selected per litter, or clustering of littermates was not adequately addressed, creating potential unit-of-analysis bias. | NR |
| 53 | Adult neurogenesis in the ventral hippocampus decreased among animal models of neurodevelopmental disorders | H | Dam/litter allocation, pups selected per litter, or clustering of littermates was not adequately addressed, creating potential unit-of-analysis bias. | 3 |
| 54 | Restoration of nNOS Expression Rescues Autistic-Like Phenotypes Through Normalization of AMPA Receptor-Mediated Neurotransmission | H | Prenatally exposed offspring were analyzed without clear dam/litter allocation or litter-level control. | - |
| 55 | Low-frequency RTMS attenuates social impairment in the VPA-induced mouse model | H | Prenatally exposed offspring were analyzed without clear dam/litter allocation or litter-level control. | - |
| 56 | Supplementation with stigma maydis polysaccharide attenuates autism‐like behaviors and improves gut function in valproic acid‐induced autism model male rats | H | Offspring were analyzed without clear statistical control for litter clustering, creating potential pseudoreplication bias. | 2 |
| 57 | The Impact of Astroglia Kir4.1 Channel Dysfunction on Neuronal Activity and Autism‐Related Behavioral Abnormalities | H | Offspring were analyzed without clear statistical control for litter clustering, creating potential pseudoreplication bias. | 2 |
| 58 | BDNF and GSK-3beta expression changes underlie the beneficial effects of crocin on behavioral alterations in a rat model of autism induced by prenatal valproic acid administration | H | Prenatally exposed offspring were analyzed without clear dam/litter allocation or litter-level control. | - |
| 59 | Microglia inversely regulate the level of perineuronal nets with the treatment of lipopolysaccharide and valproic acid | U | Random sequence generation was not reported. | - |
| 60 | Vitamin C and Gallic Acid Ameliorate Motor Dysfunction, Cognitive Deficits, and Brain Oxidative Stress | H | Dam/litter allocation, pups selected per litter, or clustering of littermates was not adequately addressed, creating potential unit-of-analysis bias. | 3 |
| 61 | Taprenepag restores maternal–fetal interface homeostasis for the treatment of neurodevelopmental disorders | H | Offspring were analyzed without clear statistical control for litter clustering, creating potential pseudoreplication bias. | 1 |
| 62 | Lipopolysaccharide induces neuroinflammation in a valproic acid male model of autism | U | Random assignment was stated, but the sequence-generation method was not described. | 2 |
| 63 | Reversal of electrophysiological and behavioral deficits mediated by 5-HT7 receptor upregulation following LP-211 treatment in an autistic-like rat model induced by prenatal valproic acid exposure | H | Prenatally exposed offspring were analyzed without clear dam/litter allocation or litter-level control. | - |
| 64 | NEAT1 Promotes Valproic Acid-Induced Autism Spectrum Disorder by Recruiting YY1 to Regulate UBE3A Transcription | H | Offspring were analyzed without clear statistical control for litter clustering, creating potential pseudoreplication bias. | 2 |
| 65 | A critical period for paired-housing-dependent autistic-like behaviors attenuation | H | Dam/litter allocation, pups selected per litter, or clustering of littermates was not adequately addressed, creating potential unit-of-analysis bias. | 1 |
| 66 | Soluble epoxide hydrolase deletion rescues behavioral and synaptic deficits by AMPK-mTOR pathway in autism animals | H | Offspring were analyzed without clear statistical control for litter clustering, creating potential pseudoreplication bias. | 2 |
| 67 | Pairing tones with vagus nerve stimulation improves brain stem responses to speech in the valproic acid model of autism | H | Offspring were analyzed without clear statistical control for litter clustering, creating potential pseudoreplication bias. | 2 |
| 68 | Adolescent treadmill exercise enhances hippocampal brain-derived neurotrophic factor (BDNF) expression and improves cognition in autism-modeled rats | H | Offspring were analyzed without clear statistical control for litter clustering, creating potential pseudoreplication bias. | 4 |
| 69 | tVNS alters inflammatory response in adult VPA ‐induced mouse model of autism: evidence for sexual dimorphism | H | Offspring were analyzed without clear statistical control for litter clustering, creating potential pseudoreplication bias. | 1 |
| 71 | Increased Serotonin Levels and Unchanged Glutamate and GABA Levels in Thalamic Microdialysates Despite Reduced Cell Numbers in a Valproic Acid-Induced Autism Model | H | Offspring were analyzed without clear statistical control for litter clustering, creating potential pseudoreplication bias. | 2 |
| 72 | Investigating brain–gut microbiota dynamics and inflammatory processes in an autistic-like rat model using MRI biomarkers during childhood and adolescence | H | Offspring were analyzed without clear statistical control for litter clustering, creating potential pseudoreplication bias. | 3 |
| 73 | Spontaneous Calcium Transients Recorded from Striatal Astrocytes in a Preclinical Model of Autism | H | Offspring were analyzed without clear statistical control for litter clustering, creating potential pseudoreplication bias. | 2 |
| 74 | Acetyl-l-carnitine alleviates valproate-induced autism-like behaviors through attenuation of hippocampal mitochondrial dysregulation | H | Offspring were analyzed without clear statistical control for litter clustering, creating potential pseudoreplication bias. | 2 |
| 75 | Striatal Interneuron Imbalance in a Valproic Acid-Induced Model of Autism in Rodents | H | Dam/litter allocation, pups selected per litter, or clustering of littermates was not adequately addressed, creating potential unit-of-analysis bias. | 2 |
| 76 | Altered prefrontal and cerebellar parvalbumin neuron counts are associated with cognitive changes in male rats | U | Random sequence generation was not reported. | - |
| 77 | Region-Specific Brain Volume Changes Emerge in Adolescence in the Valproic Acid Model of Autism and Parallel Human Findings | H | Offspring were analyzed without clear statistical control for litter clustering, creating potential pseudoreplication bias. | 2 |
| 78 | Obliteration of a glycinergic projection to the medial geniculate in an animal model of autism | H | Dam/litter allocation, pups selected per litter, or clustering of littermates was not adequately addressed, creating potential unit-of-analysis bias. | 2 |
| 79 | Construction of an animal model of autism based on interaction between cerebellar histological, immunohistochemical, and biochemical changes in adult male albino rat | H | Offspring were analyzed without clear statistical control for litter clustering, creating potential pseudoreplication bias. | 3 |
| 80 | Pharmacological intervention of behavioural traits and brain histopathology of prenatal valproic acid-induced mouse model of autism | H | Dam/litter allocation, pups selected per litter, or clustering of littermates was not adequately addressed, creating potential unit-of-analysis bias. | NR |
| 81 | Prenatal exposure to valproic acid reduces synaptic δ-catenin levels and disrupts ultrasonic vocalization in neonates | H | Offspring were analyzed without clear statistical control for litter clustering, creating potential pseudoreplication bias. | 7 |
| 82 | Prenatal exposure to valproic acid induces sex-specific alterations in rat cortical and hippocampal neuronal structure and function in vitro | H | Offspring were analyzed without clear statistical control for litter clustering, creating potential pseudoreplication bias. | 2 |
| 83 | Salidroside ameliorates neuroinflammation in autistic rats by inhibiting NLRP3/Caspase-1/GSDMD signal pathway | H | Offspring were analyzed without clear statistical control for litter clustering, creating potential pseudoreplication bias. | 1 |
| 84 | Gastrointestinal problems in a valproic acid-induced rat model of autism: From maternal intestinal health to offspring intestinal function | H | Offspring were analyzed without clear statistical control for litter clustering, creating potential pseudoreplication bias. | 3 |
| 85 | Continuous exercise training rescues hippocampal long-term potentiation in the VPA rat model of Autism: Uncovering sex-specific effects | H | Offspring were analyzed without clear statistical control for litter clustering, creating potential pseudoreplication bias. | 1 |
| 86 | Aerobic exercise improves cognitive flexibility and modulates regional volume changes in a rat model of autism | H | Offspring were analyzed without clear statistical control for litter clustering, creating potential pseudoreplication bias. | 3 |
| 87 | KCNH5 deletion increases autism susceptibility by regulating neuronal growth through Akt/mTOR signaling pathway | H | Offspring were analyzed without clear statistical control for litter clustering, creating potential pseudoreplication bias. | 6 |
| 88 | Gestational VPA exposure reduces the density of juxtapositions between TH+ axons and calretinin or calbindin expressing cells | U | Sequence generation was not explicitly reported. | NR |

**Supplementary Table S3. ARRIVE 2.0 reporting-completeness judgments**

| **Ref** | **Study** | **A1** | **A2** | **A3** | **A4** | **A5** | **A6** | **A7** | **A8** | **A9** | **A10** | **A11** | **A12** | **A13** | **A14** | **A15** | **A16** | **A17** | **A18** | **A19** | **A20** | **A21** | **Y** | **P** | **N** |
| --- | --- | --- | --- | --- | --- | --- | --- | --- | --- | --- | --- | --- | --- | --- | --- | --- | --- | --- | --- | --- | --- | --- | --- | --- | --- |
| 19 | Mechanistic insights into retinoic-acid treatment for autism in the improvement of social behavior: Evidence from a multi omics study in rats | P | P | N | N | P | P | Y | Y | Y | P | Y | Y | P | Y | P | P | Y | P | N | P | Y | 8 | 10 | 3 |
| 21 | Promising therapeutic effects of high-frequency repetitive transcranial magnetic stimulation (HF-rTMS) in addressing autism spectrum disorder induced by valproic acid | P | P | N | P | N | P | P | P | Y | P | Y | Y | Y | Y | P | P | Y | P | N | P | Y | 7 | 11 | 3 |
| 22 | Perindopril Ameliorates Sodium Valproate-Induced Rat Model of Autism | P | P | N | P | N | P | P | P | Y | P | Y | Y | Y | Y | P | N | Y | P | N | P | Y | 7 | 10 | 4 |
| 23 | Genetic machinery which accompanies metaplasticity operates differentially in experimental model of autism | P | P | N | N | P | P | N | Y | Y | P | Y | Y | P | Y | P | P | Y | P | N | P | Y | 7 | 10 | 4 |
| 24 | Animal Model of Autism Induced by Valproic Acid Combined with Maternal Deprivation: Sex-Specific Effects on Inflammation and Oxidative Stress | P | P | N | N | N | P | N | Y | Y | P | Y | Y | P | Y | Y | P | Y | P | N | P | Y | 8 | 8 | 5 |
| 25 | Prenatal Valproic Acid Induces Autistic‐Like Behaviors in Rats via Dopaminergic Modulation in Nigrostriatal and Mesocorticolimbic Pathways | P | P | N | N | N | P | Y | Y | Y | Y | Y | Y | Y | N | Y | P | Y | Y | N | P | P | 10 | 6 | 5 |
| 26 | VPA-induced autism impairs memory ability through disturbing neural oscillatory patterns in offspring rats | P | P | N | P | N | P | P | Y | P | P | Y | Y | Y | Y | P | P | Y | P | N | P | Y | 7 | 11 | 3 |
| 27 | Chinese acupuncture: A potential treatment for autism rat model via improving synaptic function | P | P | N | P | N | P | Y | Y | P | P | Y | Y | P | Y | P | P | Y | P | N | P | Y | 7 | 11 | 3 |
| 28 | Effects of Cannabidiol Isolated or in Association With Risperidone in an Animal Model of Autism | P | P | N | P | N | P | Y | Y | Y | P | Y | Y | Y | N | P | P | Y | P | N | N | P | 7 | 9 | 5 |
| 29 | The potential ameliorative effect of mesenchymal stem cells-derived exosomes on cerebellar histopathology and their modifying role on PI3k-mTOR signaling in rat model of autism spectrum disorder | P | P | N | N | N | P | N | Y | Y | P | Y | Y | Y | N | P | P | Y | P | N | P | P | 6 | 9 | 6 |
| 30 | Integrating 16S rRNA Gene Sequencing and Metabolomics Analysis to Reveal the Mechanism of L-Proline in Preventing Autism-like Behavior in Mice | P | P | N | P | P | P | P | P | Y | P | Y | Y | Y | Y | P | P | Y | P | N | P | Y | 7 | 12 | 2 |
| 31 | Neuroprotective effects of Apigenin on prenatal Valproic acid-induced autism spectrum disorder in rats | P | P | N | P | N | P | Y | Y | Y | P | Y | Y | Y | N | Y | P | Y | P | N | P | Y | 9 | 8 | 4 |
| 32 | Comparative effect of atorvastatin and risperidone on modulation of TLR4/NF-κB/NOX-2 in a rat model of valproic acid-induced autism | P | P | N | P | P | P | P | P | Y | P | Y | Y | Y | Y | P | P | Y | P | N | N | Y | 7 | 11 | 3 |
| 33 | Embryonic exposure to environmental factors drives transmitter switching in the neonatal mouse cortex causing autistic-like adult behavior | P | P | N | N | N | P | P | Y | Y | P | Y | Y | Y | N | Y | P | Y | N | N | P | Y | 8 | 7 | 6 |
| 35 | Autistic-like behaviour and changes in thalamic cell numbers a rat model of valproic acid-induced autism; A behavioural and stereological study | P | P | P | N | N | P | N | Y | Y | P | Y | Y | Y | N | P | P | Y | P | Y | P | Y | 8 | 9 | 4 |
| 36 | Autism spectrum disorder-like behaviors induced by hyper-glutamatergic NMDA receptor signaling through hypo-serotonergic 5-HT1A receptor signaling | P | P | N | P | N | P | P | P | Y | P | Y | Y | Y | N | P | P | Y | P | N | P | Y | 6 | 11 | 4 |
| 37 | Developmental Trajectory of Autistic‐Like Behaviors in a Prenatal Valproic Acid Rat Model of Autism | P | P | N | N | N | P | Y | Y | P | P | Y | Y | P | N | P | N | Y | P | N | P | P | 5 | 10 | 6 |
| 38 | Sortilin is associated with progranulin deficiency and autism‐like behaviors in valproic acid‐induced autism rats | P | P | N | P | N | P | Y | Y | Y | P | Y | Y | Y | Y | P | Y | Y | P | N | P | Y | 10 | 8 | 3 |
| 39 | Clostridium butyricum regulates intestinal barrier function via trek1 to improve behavioral abnormalities in mice with autism spectrum disorder | P | P | N | N | N | P | N | Y | Y | Y | Y | Y | P | Y | Y | P | Y | N | N | N | Y | 9 | 5 | 7 |
| 40 | Administration of Atosiban, an oxytocin receptor antagonist, ameliorates autistic-like behaviors in a female rat model of valproic acid-induced autism | P | P | N | P | N | P | Y | Y | Y | Y | Y | Y | Y | P | Y | Y | Y | P | N | P | Y | 11 | 7 | 3 |
| 41 | Oral edaravone ameliorates behavioral deficits and pathologies in a valproic acid-induced rat model of autism spectrum disorder | P | P | N | P | N | P | P | P | Y | P | Y | Y | Y | Y | Y | P | Y | P | N | P | Y | 8 | 10 | 3 |
| 42 | Transient CSF1R inhibition ameliorates behavioral deficits in Cntnap2 knockout and valproic acid-exposed mouse models of autism | P | P | N | P | N | P | Y | Y | P | P | Y | Y | Y | P | P | P | Y | P | N | N | Y | 7 | 10 | 4 |
| 43 | Biomimetic nanoparticles with enhanced rapamycin delivery for autism spectrum disorder treatment | P | P | N | P | N | P | P | P | Y | P | Y | Y | Y | Y | P | N | Y | P | N | N | Y | 7 | 9 | 5 |
| 44 | Sigma-1 receptor modulation by fluvoxamine ameliorates valproic acid-induced autistic behavior in rats: Involvement of chronic ER stress modulation, enhanced autophagy and M1/M2 microglia polarization | P | Y | N | N | P | P | Y | Y | Y | Y | Y | Y | P | N | P | P | Y | P | N | P | P | 8 | 9 | 4 |
| 45 | Benefits of bone marrow mesenchymal stem cells compared to their conditioned medium in valproic acid-induced autism in rats | P | P | N | P | N | P | Y | Y | Y | P | Y | Y | Y | N | Y | P | Y | Y | N | P | Y | 10 | 7 | 4 |
| 46 | Brain region-specific neural activation by low-dose opioid promotes social behavior | P | P | N | P | N | P | P | P | Y | P | Y | Y | Y | Y | P | P | Y | P | N | P | Y | 7 | 11 | 3 |
| 47 | Acute cannabidiol treatment reverses behavioral impairments induced by embryonic valproic acid exposure in male mice | P | P | N | P | N | P | Y | P | P | P | Y | Y | Y | N | P | P | Y | P | N | P | Y | 6 | 11 | 4 |
| 48 | Prebiotic diet normalizes aberrant immune and behavioral phenotypes in a mouse model of autism spectrum disorder | P | P | N | N | N | P | Y | Y | Y | Y | Y | Y | Y | N | Y | P | Y | Y | N | N | Y | 11 | 4 | 6 |
| 50 | The neuroprotective effect of Diosgenin in the rat Valproic acid model of autism | P | P | N | P | N | P | P | Y | Y | P | Y | Y | Y | Y | P | N | Y | P | N | P | Y | 8 | 9 | 4 |
| 51 | An alpha 5‐GABA A receptor positive allosteric modulator attenuates social and cognitive deficits without changing dopamine system hyperactivity in rats exposed to valproic acid in utero | P | P | N | N | N | P | Y | Y | P | Y | Y | Y | P | Y | P | P | Y | P | N | N | P | 7 | 9 | 5 |
| 52 | Human umbilical cord-derived mesenchymal stem cells alleviate valproate-induced immune stress and social deficiency in rats | P | P | N | P | N | P | P | P | Y | P | Y | Y | Y | Y | P | P | Y | P | N | P | Y | 7 | 11 | 3 |
| 53 | Adult neurogenesis in the ventral hippocampus decreased among animal models of neurodevelopmental disorders | P | P | P | N | N | P | P | P | Y | P | Y | Y | Y | N | P | N | Y | P | N | P | Y | 6 | 10 | 5 |
| 54 | Restoration of nNOS Expression Rescues Autistic-Like Phenotypes Through Normalization of AMPA Receptor-Mediated Neurotransmission | P | P | N | N | N | P | P | Y | Y | P | Y | Y | P | N | P | P | Y | P | N | P | Y | 6 | 10 | 5 |
| 55 | Low-frequency RTMS attenuates social impairment in the VPA-induced mouse model | P | N | N | N | N | P | Y | Y | Y | P | Y | Y | Y | Y | Y | P | Y | Y | N | P | P | 10 | 6 | 5 |
| 56 | Supplementation with stigma maydis polysaccharide attenuates autism‐like behaviors and improves gut function in valproic acid‐induced autism model male rats | P | P | N | P | N | P | Y | Y | P | P | Y | Y | P | Y | P | N | Y | P | N | P | Y | 7 | 10 | 4 |
| 57 | The Impact of Astroglia Kir4.1 Channel Dysfunction on Neuronal Activity and Autism‐Related Behavioral Abnormalities | P | P | N | P | N | P | Y | Y | Y | P | Y | Y | Y | Y | P | P | Y | Y | N | P | P | 9 | 9 | 3 |
| 58 | BDNF and GSK-3beta expression changes underlie the beneficial effects of crocin on behavioral alterations in a rat model of autism induced by prenatal valproic acid administration | P | P | N | N | N | P | N | Y | P | P | Y | Y | Y | P | P | N | Y | P | N | P | P | 5 | 10 | 6 |
| 59 | Microglia inversely regulate the level of perineuronal nets with the treatment of lipopolysaccharide and valproic acid | P | P | N | N | N | P | Y | N | P | P | Y | Y | P | N | N | P | Y | N | N | P | Y | 5 | 8 | 8 |
| 60 | Vitamin C and Gallic Acid Ameliorate Motor Dysfunction, Cognitive Deficits, and Brain Oxidative Stress | P | P | N | P | N | P | P | P | Y | P | Y | Y | Y | Y | P | N | Y | P | N | P | Y | 7 | 10 | 4 |
| 61 | Taprenepag restores maternal–fetal interface homeostasis for the treatment of neurodevelopmental disorders | P | P | N | N | N | P | P | Y | Y | P | Y | Y | P | N | N | Y | Y | P | N | P | Y | 7 | 8 | 6 |
| 62 | Lipopolysaccharide induces neuroinflammation in a valproic acid male model of autism | Y | P | N | P | N | P | P | Y | Y | P | Y | Y | Y | Y | Y | N | Y | P | N | P | Y | 10 | 7 | 4 |
| 63 | Reversal of electrophysiological and behavioral deficits mediated by 5-HT7 receptor upregulation following LP-211 treatment in an autistic-like rat model induced by prenatal valproic acid exposure | P | P | N | N | N | P | Y | Y | Y | P | Y | Y | Y | Y | Y | P | Y | P | N | P | Y | 10 | 7 | 4 |
| 64 | NEAT1 Promotes Valproic Acid-Induced Autism Spectrum Disorder by Recruiting YY1 to Regulate UBE3A Transcription | P | P | N | P | N | P | Y | Y | Y | P | Y | Y | Y | Y | P | N | Y | P | N | P | P | 8 | 9 | 4 |
| 65 | A critical period for paired-housing-dependent autistic-like behaviors attenuation | P | P | N | P | N | P | P | P | Y | P | Y | Y | Y | N | P | P | Y | P | N | P | Y | 6 | 11 | 4 |
| 66 | Soluble epoxide hydrolase deletion rescues behavioral and synaptic deficits by AMPK-mTOR pathway in autism animals | P | P | N | N | N | P | Y | Y | P | P | Y | Y | P | Y | P | N | Y | P | N | P | Y | 7 | 9 | 5 |
| 67 | Pairing tones with vagus nerve stimulation improves brain stem responses to speech in the valproic acid model of autism | P | N | N | P | P | P | Y | Y | P | P | Y | Y | Y | Y | N | P | Y | P | N | Y | P | 8 | 9 | 4 |
| 68 | Adolescent treadmill exercise enhances hippocampal brain-derived neurotrophic factor (BDNF) expression and improves cognition in autism-modeled rats | P | N | P | P | N | P | Y | Y | Y | P | Y | Y | P | Y | N | P | Y | P | N | P | Y | 8 | 9 | 4 |
| 69 | tVNS alters inflammatory response in adult VPA ‐induced mouse model of autism: evidence for sexual dimorphism | P | P | N | P | N | P | Y | Y | Y | P | Y | Y | Y | Y | P | P | Y | Y | N | P | P | 9 | 9 | 3 |
| 71 | Increased Serotonin Levels and Unchanged Glutamate and GABA Levels in Thalamic Microdialysates Despite Reduced Cell Numbers in a Valproic Acid-Induced Autism Model | P | P | N | N | N | P | P | Y | P | P | Y | Y | Y | Y | P | P | Y | N | N | P | Y | 7 | 9 | 5 |
| 72 | Investigating brain–gut microbiota dynamics and inflammatory processes in an autistic-like rat model using MRI biomarkers during childhood and adolescence | P | P | N | N | N | P | N | Y | P | Y | Y | Y | Y | Y | Y | N | Y | P | N | P | Y | 9 | 6 | 6 |
| 73 | Spontaneous Calcium Transients Recorded from Striatal Astrocytes in a Preclinical Model of Autism | P | P | N | N | N | P | Y | Y | P | P | Y | Y | P | P | P | N | Y | P | N | P | Y | 6 | 10 | 5 |
| 74 | Acetyl-l-carnitine alleviates valproate-induced autism-like behaviors through attenuation of hippocampal mitochondrial dysregulation | P | P | P | P | N | P | N | Y | Y | P | Y | Y | Y | Y | Y | P | Y | P | N | N | Y | 9 | 8 | 4 |
| 75 | Striatal Interneuron Imbalance in a Valproic Acid-Induced Model of Autism in Rodents | P | P | N | P | N | P | P | P | Y | P | Y | Y | Y | N | P | N | Y | P | N | N | P | 5 | 10 | 6 |
| 76 | Altered prefrontal and cerebellar parvalbumin neuron counts are associated with cognitive changes in male rats | P | N | N | N | N | P | Y | Y | Y | P | Y | Y | P | Y | P | P | Y | Y | N | P | Y | 9 | 7 | 5 |
| 77 | Region-Specific Brain Volume Changes Emerge in Adolescence in the Valproic Acid Model of Autism and Parallel Human Findings | P | N | N | N | N | P | N | Y | Y | P | Y | Y | P | Y | N | P | Y | N | N | P | Y | 7 | 6 | 8 |
| 78 | Obliteration of a glycinergic projection to the medial geniculate in an animal model of autism | P | P | N | N | N | P | P | P | Y | P | Y | Y | Y | Y | P | N | Y | P | N | P | Y | 7 | 9 | 5 |
| 79 | Construction of an animal model of autism based on interaction between cerebellar histological, immunohistochemical, and biochemical changes in adult male albino rat | P | P | N | P | N | P | N | Y | Y | P | Y | Y | P | N | Y | P | Y | P | N | P | Y | 7 | 9 | 5 |
| 80 | Pharmacological intervention of behavioural traits and brain histopathology of prenatal valproic acid-induced mouse model of autism | P | P | N | P | N | P | P | P | Y | P | Y | Y | Y | Y | P | P | Y | P | N | Y | Y | 8 | 10 | 3 |
| 81 | Prenatal exposure to valproic acid reduces synaptic δ-catenin levels and disrupts ultrasonic vocalization in neonates | P | P | N | N | N | P | Y | P | P | P | Y | Y | Y | Y | P | N | Y | P | N | N | Y | 7 | 8 | 6 |
| 82 | Prenatal exposure to valproic acid induces sex-specific alterations in rat cortical and hippocampal neuronal structure and function in vitro | P | P | N | N | N | P | Y | Y | Y | P | Y | Y | P | N | Y | P | Y | P | Y | Y | Y | 10 | 7 | 4 |
| 83 | Salidroside ameliorates neuroinflammation in autistic rats by inhibiting NLRP3/Caspase-1/GSDMD signal pathway | P | P | N | N | N | P | P | P | P | P | Y | Y | Y | N | P | P | Y | P | N | P | Y | 5 | 11 | 5 |
| 84 | Gastrointestinal problems in a valproic acid-induced rat model of autism: From maternal intestinal health to offspring intestinal function | P | P | N | P | N | P | Y | Y | Y | P | Y | Y | P | Y | Y | P | Y | P | N | N | P | 8 | 9 | 4 |
| 85 | Continuous exercise training rescues hippocampal long-term potentiation in the VPA rat model of Autism: Uncovering sex-specific effects | P | P | N | P | N | P | Y | Y | Y | Y | Y | Y | P | Y | P | P | Y | P | N | N | Y | 9 | 8 | 4 |
| 86 | Aerobic exercise improves cognitive flexibility and modulates regional volume changes in a rat model of autism | P | N | N | P | N | P | P | Y | Y | P | Y | Y | P | Y | N | P | Y | P | N | P | P | 6 | 10 | 5 |
| 87 | KCNH5 deletion increases autism susceptibility by regulating neuronal growth through Akt/mTOR signaling pathway | P | P | N | N | N | P | Y | Y | Y | P | Y | Y | P | Y | Y | P | Y | P | N | P | Y | 9 | 8 | 4 |
| 88 | Gestational VPA exposure reduces the density of juxtapositions between TH+ axons and calretinin or calbindin expressing cells | P | P | N | N | P | P | P | P | Y | P | Y | Y | Y | N | P | N | Y | P | N | P | Y | 6 | 10 | 5 |

ARRIVE ratings: Y, adequately reported; P, partially reported; N, not reported. A1–A21 correspond to the ARRIVE 2.0 items listed in Supplementary Table S4.

**Supplementary Table S4. ARRIVE 2.0 item definitions and aggregate reporting**

| **Item** | **ARRIVE 2.0 domain** | **Adequate (Y)** | **Partial (P)** | **Not reported (N)** |
| --- | --- | --- | --- | --- |
| 1 | Study design | 1 | 65 | 0 |
| 2 | Sample size | 1 | 59 | 6 |
| 3 | Inclusion/exclusion criteria | 0 | 4 | 62 |
| 4 | Randomization | 0 | 35 | 31 |
| 5 | Blinding | 0 | 7 | 59 |
| 6 | Outcome measures | 0 | 66 | 0 |
| 7 | Statistical methods | 31 | 25 | 10 |
| 8 | Experimental animals | 46 | 19 | 1 |
| 9 | Experimental procedures | 50 | 16 | 0 |
| 10 | Results | 8 | 58 | 0 |
| 11 | Abstract | 66 | 0 | 0 |
| 12 | Background | 66 | 0 | 0 |
| 13 | Objectives | 43 | 23 | 0 |
| 14 | Ethical statement | 40 | 4 | 22 |
| 15 | Housing and husbandry | 18 | 42 | 6 |
| 16 | Animal care and monitoring | 3 | 46 | 17 |
| 17 | Interpretation/scientific implications | 66 | 0 | 0 |
| 18 | Generalizability/translation | 7 | 54 | 5 |
| 19 | Protocol registration | 2 | 0 | 64 |
| 20 | Data access | 3 | 51 | 12 |
| 21 | Declaration of interests | 51 | 15 | 0 |

**Overall interpretation.** Among the 66 eligible prenatal VPA studies, 62 were judged at overall high risk of bias and 4 at overall unclear risk; none met a low-risk overall classification. The most frequent limitations were incomplete reporting of allocation concealment, random housing, personnel blinding, random outcome assessment, animal-flow accounting, and litter-level experimental-unit handling. ARRIVE reporting was generally strongest for experimental procedures, abstracts, scientific background, objectives, and ethics, whereas inclusion/exclusion criteria, blinding, animal-care monitoring, protocol registration, and data-access procedures were commonly incomplete.
